# A phytobacterial TIR domain effector manipulates NAD^+^ to promote virulence

**DOI:** 10.1111/nph.17805

**Published:** 2021-11-05

**Authors:** Samuel Eastman, Thomas Smith, Mark A. Zaydman, Panya Kim, Samuel Martinez, Neha Damaraju, Aaron DiAntonio, Jeffrey Milbrandt, Thomas E. Clemente, James R. Alfano, Ming Guo

**Affiliations:** ^1^ Department of Plant Pathology University of Nebraska‐Lincoln Lincoln NE 68583 USA; ^2^ Department of Chemistry University of Nebraska‐Lincoln Lincoln NE 68583 USA; ^3^ Department of Pathology and Immunology Washington University School of Medicine St Louis MO 63110 USA; ^4^ The Center for Plant Science Innovation University of Nebraska‐Lincoln Lincoln NE 68588 USA; ^5^ School of Biological Sciences University of Nebraska‐Lincoln Lincoln NE 68583 USA; ^6^ Department of Biomedical Engineering Washington University in St Louis St Louis MO 63130 USA; ^7^ Department of Developmental Biology Washington University School of Medicine St Louis MO 63110 USA; ^8^ Department of Genetics Washington University School of Medicine St Louis MO 63110 USA; ^9^ Department of Agriculture and Horticulture University of Nebraska‐Lincoln Lincoln NE 68583 USA

**Keywords:** cADPR variant, HopAM1, NAD^+^, NAD^+^ hydrolase, *Pseudomonas syringae*, TIR domain, v2‐cADPR, v‐cADPR

## Abstract

- The *Pseudomonas syringae* DC3000 type III effector HopAM1 suppresses plant immunity and contains a Toll/interleukin‐1 receptor (TIR) domain homologous to immunity‐related TIR domains of plant nucleotide‐binding leucine‐rich repeat receptors that hydrolyze nicotinamide adenine dinucleotide (NAD^+^) and activate immunity. *In vitro* and *in vivo* assays were conducted to determine if HopAM1 hydrolyzes NAD^+^ and if the activity is essential for HopAM1’s suppression of plant immunity and contribution to virulence.
- HPLC and LC‐MS were utilized to analyze metabolites produced from NAD^+^ by HopAM1 *in vitro* and in both yeast and plants. *Agrobacterium*‐mediated transient expression and *in planta* inoculation assays were performed to determine HopAM1’s intrinsic enzymatic activity and virulence contribution.
- HopAM1 is catalytically active and hydrolyzes NAD^+^ to produce nicotinamide and a novel cADPR variant (v2‐cADPR). Expression of HopAM1 triggers cell death in yeast and plants dependent on the putative catalytic residue glutamic acid 191 (E191) within the TIR domain. Furthermore, HopAM1’s E191 residue is required to suppress both pattern‐triggered immunity and effector‐triggered immunity and promote *P. syringae* virulence.
- HopAM1 manipulates endogenous NAD^+^ to produce v2‐cADPR and promote pathogenesis. This work suggests that HopAM1’s TIR domain possesses different catalytic specificity than other TIR domain‐containing NAD^+^ hydrolases and that pathogens exploit this activity to sabotage NAD^+^ metabolism for immune suppression and virulence.

## Introduction

Toll/interleukin‐1 receptor (TIR) domain proteins are an ancient family of nicotinamide adenine dinucleotide (NAD^+^) hydrolases (Essuman *et al*., 2018). TIR domains are found across kingdoms including archaea, bacteria, animals and plants (Bayless & Nishimura, 2020). In animals, TIR domain‐containing Toll‐like receptors (TLRs) serve as pattern recognition receptors that recognize pathogen‐ or microbe‐associated molecular patterns (PAMPs or MAMPs) and activate innate immunity (O'Neill *et al*., 2013). One animal TIR domain‐containing protein, the highly conserved sterile alpha and TIR motif containing 1 (SARM1), serves as a TLR inhibitor and possesses NAD^+^ hydrolase activity that causes NAD^+^ depletion and neuronal cell death (Essuman *et al*., 2017; Carty & Bowie, 2019). In plants, TIR domain nucleotide‐binding leucine‐rich repeat receptors (TIR‐NLRs), together with coiled‐coil (CC)‐domain NLRs, make up the two major groups of intracellular receptors that activate immunity and serve as resistance proteins (R proteins) (Ma *et al*., 2020; Martin *et al*., 2020). Recently, it was found that direct or indirect recognition of pathogenic effectors in plants results in oligomerization of R‐gene NLRs mediated by the CC domain or TIR domain and this oligomerization of TIR‐NLRs leads to NAD^+^ cleavage that results in immune activation and cell death (Horsefield *et al*., 2019; Wan *et al*., 2019; Ma *et al*., 2020; Martin *et al*., 2020).

TIR domain‐containing proteins (Tcps) are widely distributed in prokaryotes but more sparsely in fungi and viruses and have been classified as a family of virulence factors, some of which disrupt TLR signaling in animal hosts (Bowie *et al*., 2000; Newman *et al*., 2006; Spear *et al*., 2009; Cirl & Miethke, 2010; Alaidarous *et al*., 2014; Patterson *et al*., 2014; Rosadini *et al*., 2015; Imbert *et al*., 2017). Prokaryotic TIR domain proteins can also hydrolyze NAD^+^, including TIR domain‐containing effectors BtpA and BtpB from the animal pathogen *Brucella abortus* that deplete host NAD^+^ while suppressing TLR signaling (Essuman *et al*., 2018; Coronas‐Serna *et al*., 2020). TIR domains present in a wide range of microbial genomes are probable components of prokaryotic antiviral immunity (Ma *et al*., 2020; Ofir *et al*., 2021). To date, no TIR domain‐containing effectors of phytopathogens have been reported to hydrolyze NAD^+^. A few non‐TIR domain effectors use NAD^+^ as a substrate for their immune‐suppressive activity: *Pseudomonas syringae* effectors HopU1 and HopF2 utilize NAD^+^ to ADP‐ribosylate host proteins and *Xanthomonas* effector AvrRxo1 phosphorylates NAD^+^ into NADP^+^ (Fu *et al*., 2007; Wang *et al*., 2010; Jeong *et al*., 2011; Shidore *et al*., 2017). Despite their similarity to eukaryotic TIR domains, bacterial TIR domains and their resulting cleavage products do not appear to activate plant immunity when expressed *in planta* (Duxbury *et al*., 2020). However, the influence of TIR domain effectors and NAD^+^ on the immune system remains unclear in plants.

Nicotinamide adenine dinucleotide is an essential dinucleotide molecule at the center of cellular metabolism and redox signaling (Noctor, 2006). In plants, NAD^+^ is either produced *de novo* using aspartate or via the salvage pathway using nicotinamide (Nam), nicotinamide mononucleotide or other intermediates (Gakière *et al*., 2018). NAD^+^ is functional across a range of diverse cellular activities including calcium signaling, ADP‐ribosylation and histone modification (Hunt *et al*., 2004). Although the role of NAD^+^ during pathogen infection is not fully understood, NAD^+^ is clearly implicated in a vital defense role in plant–pathogen interactions (Pétriacq *et al*., 2013, 2016). In barley, NAD^+^ content spiked in leaves infected with powdery mildew (Ryrie & Scott, 1968). Overproduction of endogenous NAD^+^ in Arabidopsis stimulated the salicylic acid pathway leading to enhanced resistance to the avirulent strain *Pto*‐AvrRpm1 (Pétriacq *et al*., 2012). Extracellular NAD^+^ is suggested to be an elicitor of plant immunity and a possible component of plant systemic acquired resistance (Wang *et al*., 2017). Phosphorylation of NAD^+^ by *Xanthomonas* effector AvrRxo1 mitigates the reactive oxygen species (ROS) burst component of the immune response in tobacco (Shidore *et al*., 2017). Recently, NAD^+^ hydrolysis and the derived products were associated with the cell death response to pathogens in animals and plants (Horsefield *et al*., 2019; Wan *et al*., 2019). Variable cleavage of NAD^+^ by TIR domain proteins produces nicotinamide and adenine diphosphate ribose (ADPR), cyclic adenine diphosphate ribose (cADPR) or a structurally unidentified cADPR variant (v‐cADPR) with equal mass theorized to be an alternative cyclic linkage (Essuman *et al*., 2017, 2018; Wan *et al*., 2019; Duxbury *et al*., 2020). cADPR is an adenine nucleotide cyclized at the N1 position of the adenine ring that acts as a second messenger of ABA and as a mediator of calcium (Ca^2+^) release (Lee, 1994; Wu *et al*., 1997). A clear picture has yet to emerge describing the function of NAD^+^, cADPR and v‐cADPR during pathogenic interactions.

To successfully colonize hosts, pathogens must circumvent immunity. Hosts are capable of recognizing PAMPs or MAMPs and activating pattern‐triggered immunity (PTI) to restrict pathogen growth (Zipfel, 2014). In plants, effector recognition by intracellular CC‐NLRs and TIR‐NLRs potentiates a more intense effector‐triggered immunity (ETI) resulting in a hypersensitive response (HR) and plant cell death (Wu *et al*., 2017; Wan *et al*., 2019). To combat immunity, many bacterial pathogens utilize secretion systems like the type III secretion system (T3SS) to deliver effectors that suppress PTI and/or ETI by interfering with host signaling or protein components of plant immunity (Guo *et al*., 2009; Buttner, 2012; Macho & Zipfel, 2015). Pathogenic effectors possess diverse enzymatic activity and target a broad range of host physiological processes as part of a concerted effort to disrupt the immune response (Toruno *et al*., 2016). Most described effectors target host proteinaceous components but a few are thought to indirectly alter specific plant metabolites: *P*. *syringae* effector HopZ1 suppresses the isoflavone biosynthesis enzyme GmHID1 (Zhou *et al*., 2011), whereas the maize pathogen *Pantoea stewartii* effector WtsE upregulates the shikimate and phenylpropanoid pathways (Asselin *et al*., 2015). The specific mechanisms of these effectors remain unclear and their effects appear to promote virulence only indirectly (Macho, 2016). Only a few effectors directly alter specific metabolites: *Ralstonia solanacearum* effector RipAY exhibits γ‐glutamyl cyclotransferase (GGCT) activity and degrades intracellular glutathione, another *R*. *solanacearum* effector RipTPS synthesizes the plant signaling metabolite trehalose‐6‐phosphate and *Xanthomonas* type III effector AvrRxo1 phosphorylates NAD^+^ into NADP^+^ (Poueymiro *et al*., 2014; Fujiwara *et al*., 2016; Shidore *et al*., 2017). The products of these effectors, however, are metabolites already endogenous to plant cells. Little is known about how effectors directly mediate conversion of host metabolites into novel compounds or about the consequences of this activity on plant immunity and virulence.

*Pseudomonas syringae* is a hemibiotrophic bacterial pathogen with pathovars that can infect many plant species, including crops of agronomic importance. *P. syringae* pv. *tomato* (*Pto*) DC3000 is pathogenic in the model plants tomato and Arabidopsis (Xin & He, 2013). *Pto* DC3000 injects > 30 type III effectors, of which key effectors have been identified as immunity suppressors and contribute to pathogenicity (Jamir *et al*., 2004; Guo *et al*., 2009; Wei *et al*., 2015; Wei & Collmer, 2018). While significant progress has been made towards understanding the virulence contribution and host targets of *P. syringae* effectors, the catalytic activity of the majority of effectors in plants is unknown (Block & Alfano, 2011). One such effector is HopAM1, which strongly suppresses plant immunity and manipulates responses to ABA (Jamir *et al*., 2004; Goel *et al*., 2008; Guo *et al*., 2009). Ectopic expression of HopAM1 is toxic to yeast (Munkvold *et al*., 2008). Unusually, resistance to *Pto* DC3000 expressing HopAM1 appears to be polygenic instead of monogenic, and the presence of HopAM1 restricts growth of *Pto* DC3000 on some Arabidopsis ecotypes (Iakovidis *et al*., 2016; Velásquez *et al*., 2017). HopAM1 is one of a minimal set of effectors sufficient to restore virulence and disease symptoms of the poly‐effector mutant DC3000 D28E (Cunnac *et al*., 2011). Here we show that HopAM1 contains a noncanonical TIR domain and has enzymatic activity that hydrolyzes the essential metabolite NAD^+^ in plants to promote virulence.

## Materials and Methods

### Bacterial cultures, DNA manipulation and purification of recombinant proteins

Bacterial cultures, growth conditions, plasmid assembly and protein purification are described in Supporting Information Methods S1. The plasmids and strains used in this study are listed in Table S1.

### Generation of *Pto* DC3000 mutants

The *Pto* DC3000 mutants studied were generated by an unmarked mutagenesis strategy with modification (House *et al*., 2004) and are described in Methods S1.

### Plant materials

Arabidopsis plants were grown at 24°C with a 10 h : 14 h, light : dark cycle in microclimate‐controlled growth chambers. *Nicotiana benthamiana* and *Nicotiana tabacum* cv Xanthi plants were grown under standard glasshouse conditions.

### *Agrobacterium*‐mediated transient assays

*Agrobacterium* strains carrying binary vectors were infiltrated into *N. benthamiana* or *N. tabacum* leaves at an OD_600_ of 0.8. A detailed methodology is described in Methods S1.

### HPLC

For samples extracted using perchloric acid, 90 µl of sample was mixed with 10 µl of 0.5 M phosphate buffer (pH 7.0). Metabolites were analyzed using an Agilent 1260 HPLC system (Agilent, Santa Clara, CA, USA) with an Agilent Poroshell 120 EC‐C18 column (4.6 mm × 150 mm, 2.7 µm) under isocratic conditions. The mobile phase consisted of 0.05 M potassium phosphate buffer (pH 7.0) and methanol (95 : 5, v/v) with a flow rate of 1.0 ml min^−1^. The eluent was monitored at 260 nm by a diode array detector. Alternatively, *in vitro* samples and standards were reconstituted in 0.05 M phosphate buffer (pH 7.0) and analyzed using an Agilent Nexera LC‐40 with a Kinetex C18 column (3 mm × 100 mm, 2.6 µM) under isocratic conditions. The mobile phase consisted of 0.05 M potassium phosphate buffer (pH 7.0).

For sample extracted using the methanol–chloroform method, lyophilized powder was resuspended in 100 µl of 0.05 M ammonium acetate buffer (pH 6.7). Metabolites were analyzed using an Agilent 1100 HPLC system with an Agilent Poroshell 120 EC‐C18 column (4.6 mm × 150 mm, 2.7 µm) under isocratic conditions. The mobile phase consisted of 0.05 M ammonium acetate buffer (pH 6.7) and methanol (95 : 5, v/v) with a flow rate of 1.0 ml min^−1^. The eluent was monitored at 260 nm by a variable‐wavelength detector. Standards NAD^+^ (Selleck Chemicals, Pittsburgh, PA, USA), Nam (Supelco, Bellefonte, PA, USA), and cADPR (Cayman Chemical, Ann Arbor, MI, USA) were run concurrently. HPLC chromatograms were prepared using OpenChrom (https://lablicate.com/platform/openchrom).

### Mass spectrometry

Lyophilized powder was resuspended in 100 µl of 0.05 M ammonium acetate buffer. A sample of 100 µl was loaded and standards NAD^+^, Nam and cADPR were run concurrently. Metabolites were separated using a Waters NanoAcquity UPLC system with a Waters 1.0 × 50 mm column (1.8 μm packing HSS) under isocratic conditions. The mobile phase consisted of 0.05 M ammonium acetate buffer (pH 6.7) and methanol (95 : 5, v/v) with a flow rate of 40 µl min^−1^. Metabolites were analyzed using a Waters Xevo G2‐XS QTOF with electrospray ionization. MS and MS/MS chromatograms were prepared using MassLynx (https://www.waters.com/waters/en_US/MassLynx‐MS‐Software/nav.htm?cid=513662).

### *In vitro* NAD hydrolase and enzyme kinetics assays

Protocols previously reported were followed (Essuman *et al*., 2017) and are described in Methods S1.

### Yeast metabolite analysis

Yeast overnight cultures grown in liquid dropout medium Glucose‐His were centrifuged and washed with double distilled H_2_O and resuspended with Galactose‐His adjusted to an OD_600_ of 0.5. At the indicated time points, a specified volume of culture was removed and spun down for 30 s at 15 000 ***g***. For HPLC analysis, 1 ml of culture was resuspended in 200 µl ice‐cold 0.5 M perchloric acid and incubated on ice for 10 min. Samples were then subjected to three cycles of freezing in liquid nitrogen and thawing on ice. Samples were then centrifuged at 15 000 ***g*** for 5 min, and 180 µl of supernatant was then mixed with 67 µl of 3 M K_2_CO_3_ and incubated for 10 min on ice. Samples were centrifuged again at 15 000 ***g*** for 5 min and 90 µl of supernatant was saved at −80°C until HPLC analysis, whereupon the samples were amended with 10 µl of 0.5 M phosphate buffer (26.2 g l^−1^ potassium phosphate monobasic, 53.5 g l^−1^ potassium phosphate dibasic).

For MS analysis, 1 ml culture was resuspended in 250 µl of 50% methanol at −40°C and vortexed, then mixed with 250 µl chloroform at −40°C. Samples were subjected to three freeze–thaw cycles alternating between liquid nitrogen and a −40°C ethanol bath. Samples were then centrifuged at 15 000 ***g*** for 5 min at −10°C, then the aqueous/methanol layer was removed, lyophilized and stored at −80°C until MS analysis.

### Plant metabolite analysis

*Nicotiana benthamiana* leaf disks were excised 24 h after induction using a 16 mm diameter cork borer. Samples were frozen in liquid nitrogen and ground with a plastic pestle. The frozen powder was suspended in 500 µl of 50% methanol at −40°C and vortexed, then mixed with 500 µl chloroform at −40°C. Samples were subjected to two additional freeze–thaw cycles alternating between liquid nitrogen and a −40°C ethanol bath. Samples were then spun at 15 000 ***g*** for 5 min at −10°C, then the aqueous/methanol layer was removed, lyophilized and stored at −80°C until HPLC.

Overnight cultures of *Pto* DC3000 strains resuspended in 10 mM MgCl_2_ were infiltrated with a blunt syringe into leaves of 4‐wk‐old *Arabidopsis* plants. *Pto* DC3000 derivatives were infiltrated at a density of 1 × 10^5^ colony‐forming units (CFU) ml^−1^ while *Pto* DC3000D28E derivatives were infiltrated at 1 × 10^8^ CFU ml^−1^. Plants were kept in covered trays at room temperature. Infiltrated leaves were removed at the indicated times, weighed, frozen in liquid nitrogen and ground with a plastic pestle. The metabolites were extracted following the above protocol for *N. benthamiana*.

### ROS assay

Production of ROS was determined following a previously described protocol (Asai *et al*., 2008). A detailed methodology is described in Methods S1.

### Callose deposition assay

A previously described method was used for callose deposition assays (Adam & Somerville, 1996). A detailed methodology is described in Methods S1.

### *In planta* bacterial growth assay

Pathogenicity assays were carried out as previously described (Guo *et al*., 2016). A detailed methodology is described in Methods S1.

## Results

### Conserved residues within HopAM1’s putative TIR domain are required to induce cell death in yeast and plants

**Fig. 1:**
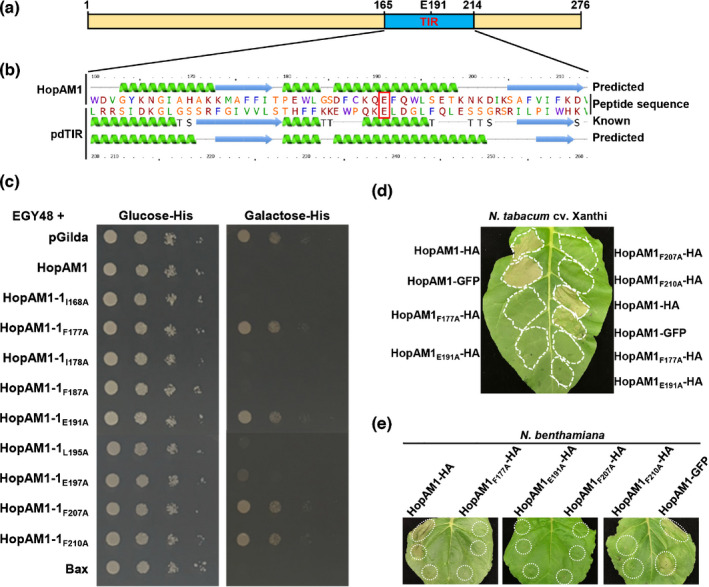
Conserved residues in HopAM1’s Toll/interleukin‐1 receptor (TIR) domain are required to trigger cell death in yeast and plants. (a) A schematic of the HopAM1 protein indicating its putative TIR domain shaded in blue and the putative catalytic glutamic acid 191 (E191). (b) Alignment of the Phyre2‐predicted secondary structure of HopAM1’s putative TIR domain with the known structure of pdTIR (PDB ID: 3H16) from *Paracoccus denitrificans*. The consensus putative catalytic glutamic acid residues are indicated in a red box. HopAM1 contains similar secondary structure to the pdTIR structure template with highest confidence in Phyre2 searches. (c) Yeast cell death assay with strains expressing HopAM1 wild‐type and mutants of conserved residues within the TIR domain. The conserved residues F177, E191, F207 and F210 were essential for HopAM1‐associated yeast cell death. Yeast EGY48 strains harboring plasmids containing *hopAM1* and the mutants of conserved residues shown in Supporting Information Fig. S1(b), pGilda (vector) and *Bax* (positive control of cell death) were induced with galactose and assessed for survival. (d) HopAM1 triggers hypersensitive response‐like cell death in *Nicotiana tabacum* cv Xanthi and (e) *Nicotiana benthamiana*. *In planta* transient expression of HopAM1 and the indicated mutations except HopAM1‐GFP were estradiol‐inducible and mediated via *Agrobacterium tumefaciens* at an OD_600_ of 0.8. Images shown in (c–e) were taken 3 d after plating yeast cultures or inducing the expression with estradiol at 20 μM in plant leaves.

Two identical *hopAM1* alleles, each encoding a 276 amino acid protein, are present in the *Pto* DC3000 genome. *hopAM1‐1* is located in the chromosome while *hopAM1‐2* is found on the endogenous plasmid pDC3000A (Buell *et al*., 2003). Homologs of HopAM1 are sporadically distributed in multiple phytopathogenic strains, predominantly in *Pseudomonas* species but also in *Xanthomonas* spp. and *Ralstonia* spp. (Fig. S1a). HopAM1 is highly similar (> 95% identity in peptide sequence) among *Pseudomonas* species but less similar among *Xanthomonas* spp. (50% identity and 68% similarity) or *Ralstonia* spp. (54% identity and 71% similarity) (Fig. S1a). The primary peptide sequence of HopAM1 does not share significant similarity with proteins with known functional domains based on an iterative Psi‐Blast (Position‐Specific Iterated Blast) analysis. We then conducted a search for proteins with structural similarity to HopAM1 using the Phyre2 algorithm for structural modeling (Kelley *et al*., 2015). A short stretch of HopAM1 peptide sequence (amino acids 165–214) was predicted to share secondary structure with known TIR domains, including those found in animal bacterial pathogens, animal TLR receptors and resistance proteins of plants associated with immunity (Fig. 1a,b) (Cirl & Miethke, 2010). Within this region of similarity, we observed multiple highly conserved amino acid residues in HopAM1 homologs from phytobacterial pathogens, including I168, F177, I178, F187, E191, L195, E197, F207 and F210 (Fig. S1b). All known enzymatically active TIR domains contain a consensus glutamic acid residue essential for NAD^+^ hydrolase catalytic activity (Essuman *et al*., 2017, 2018). Among the conserved residues, E191 was invariant among all aligned proteins, implicating it as the putative catalytic residue. Phylogenetically, the homologs present in phytopathogenic bacteria form a distinctive clade from others, suggesting the existence of a lineage of noncanonical TIR domains (Fig. S1c).

Previously, it was shown that overexpression of HopAM1 is lethal to yeast (*Saccharomyces cerevisiae*) (Jamir *et al*., 2004; Munkvold *et al*., 2008). To further examine HopAM1’s ability to induce lethality in yeast, site‐directed mutants were generated in the coding sequence of conserved residues within the putative TIR domain and cloned into pGilda. Expression of HopAM1 mutants was induced on galactose‐containing medium and all the HopAM1 mutants appeared to be stable (Fig. S1d). Strains expressing the mutants HopAM1_F177A_, HopAM1_E191A_, HopAM1_F207A_ and HopAM1_F210A_ no longer caused cell death (Fig. 1a), demonstrating that these residues are essential for the function of HopAM1’s putative TIR domain and cell death in yeast.

HopAM1 induces cell death in both *N. tabacum* and *N. benthamiana* (Choi *et al*., 2017). We next examined whether HopAM1‐associated cell death is also dependent on the same required conserved residues in yeast. HopAM1 and mutants HopAM1_F177A_, HopAM1_E191A_, HopAM1_F207A_ and HopAM1_F210A_ fused with either a hemagglutinin (HA) epitope tag or green fluorescence protein (GFP) were transiently expressed via *Agrobacterium*‐mediated transformation in *N. tabacum* cv Xanthi and *N. benthamiana* and assessed for their ability to induce cell death (Fig. S1e). Mutations of these essential TIR domain residues including the putative catalytic E191 abolished HopAM1‐induced cell death in both *N. tabacum* cv Xanthi and *N. benthamiana*. In line with cell death induction by HopAM1 in yeast, the results are consistent with the hypothesis that HopAM1’s putative TIR domain and its associated NAD^+^ hydrolase activity are required for HopAM1‐induced cell death (Fig. 1c–e).

### HopAM1 is an active NAD^+^ hydrolyzing enzyme

**Fig. 2:**
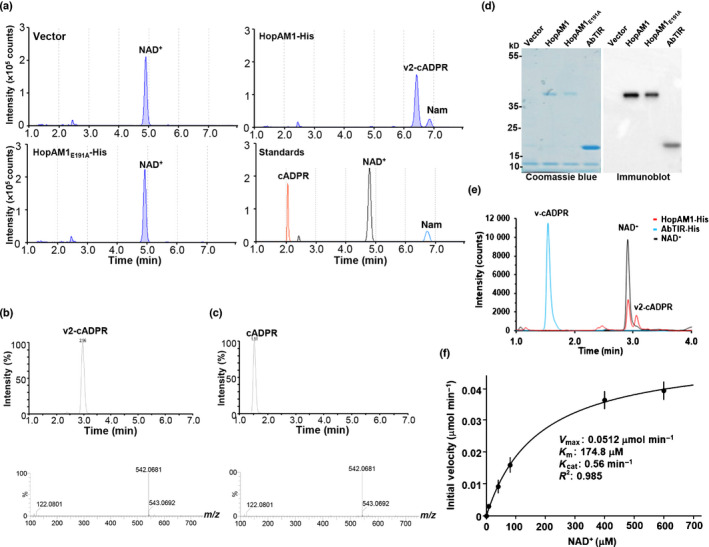
HopAM1 hydrolyzes nicotinamide adenine dinucleotide (NAD^+^) *in vitro* to produce a novel product. (a) *In vitro* assays with purified HopAM1‐His, HopAM1_E191A_‐His and mock preparation of empty vector control, and HPLC chromatographs with NAD^+^, cADPR and nicotinamide (Nam) standards. (b) Mass spectra of v2‐cADPR and (c) cADPR standard. v2‐cADPR and cADPR appeared to have the same mass but different retention time. (d) Coomassie staining and immunoblot of purified HopAM1, HopAM1_E191A_ and AbTIR. (e) Overlaid HPLC chromatographs of NAD^+^ standard and *in vitro* products of HopAM1‐His and AbTIR‐His. (f) Enzymatic kinetics of HopAM1. Purified protein and substrate NAD^+^ at a series of concentration of 1–600 μM were incubated at room temperature for 60 min. Bars indicate standard error (SE). The reaction products were extracted, analyzed by HPLC and used to calculate the rate of initial NAD^+^ consumption. In (e), the unique product v2‐cADPR is present specifically in the HopAM1 partial hydrolysis at a retention time of *c*. 3.1 min while v‐cADPR is present at 1.6 min.

TIR domains of prokaryotic and eukaryotic origins, including animal SARM1 and plant TIR‐containing NLRs, have been demonstrated to possess enzymatic activity that hydrolyzes NAD^+^ (Essuman *et al*., 2017, 2018; Horsefield *et al*., 2019; Wan *et al*., 2019). To determine whether HopAM1 hydrolyzes NAD^+^, we examined its NAD^+^ hydrolase activity *in vitro*. *hopAM1* and *hopAM1_E191A_* were cloned into pET28a(+) and the recombinant His‐tagged proteins were purified. Purified HopAM1‐His and HopAM1_E191A_‐His proteins were incubated with 60 µM NAD^+^. The reaction products were analyzed with reversed‐phase HPLC. Only input NAD^+^ substrates were observed with all reactions at time 0 (Fig. S2a). Two peaks were detected in the products from the reaction with HopAM1‐His proteins with retention time of *c*. 6.5 and 6.8 min, respectively, while the substrate NAD^+^ peak was almost exhausted, indicative of enzymatic NAD^+^ hydrolase activity (Fig. 2a). By contrast, reactions with HopAM1_E191A_‐His and the vector control did not result in any detectable peaks corresponding to the products other than the input substrate NAD^+^ (Fig. 2a). The peak with a retention time at 6.5 min was distinct from NAD^+^, cADPR and Nam standards (Fig. 2a), suggesting it is a unique product.

To identify this product, we performed LC‐MS time of flight (TOF) analysis on extracted metabolites from the *in vitro* reactions (Fig. S2b). The product was determined to have a mass of 542.0681 – identical to that of the cADPR standard. However, HopAM1’s product and the cADPR standard eluted at different times, indicating this product is a variant compound with different chemical properties from the canonical cADPR (Fig. 2b,c). The masses of Nam and remaining NAD^+^ from the *in vitro* reaction were also determined (Fig. S2c,d). Taken together, HPLC and LC‐MS analysis of the products of *in vitro* reactions confirmed HopAM1 is an active NAD^+^ hydrolyzing enzyme and the putative E191 residue within its TIR domain is required for catalytic activity.

A subset of prokaryotic TIR domains including AbTIR, a Tcp from the opportunistic bacterial pathogen *Acinetobacter baumanni*, generate a variant of cADPR hypothesized to be an alternative cyclization designated v‐cADPR (Essuman *et al*., 2018). To identify HopAM1’s unique *in vitro* product, we compared it with AbTIR’s known product via HPLC. NAD^+^ hydrolase reactions were conducted with purified HopAM1 and AbTIR (Fig. 2d). The HPLC chromatographs of their products revealed that HopAM1’s unique *in vitro* product eluted at a different time from v‐cADPR produced by AbTIR, indicating that HopAM1 hydrolyzes NAD^+^ into a product clearly distinct from v‐cADPR and designated and hereafter referred to as v2‐cADPR (Fig. 2e). HopAM1’s enzymatic properties were further determined with Michaelis–Menten kinetic assays and found to possess a Michaelis constant (*K*_m_) of 174.8 μM, a maximum velocity (*V*_max_) of 0.0512 μM min^−1^ and a catalyst rate constant (*K*_cat_) of 0.56 min^−1^ (Fig. 2f). HopAM1’s *K*_m_ (174.8 μM) is comparable to that of TirS‐TIR (490 μM) and TcpC‐TIR (196 μM) but much higher than that of SARM1 (24 μM), which is close to the intracellular NAD^+^ concentrations of 200–2000 μM in plants (Essuman *et al*., 2017, 2018; Gakière *et al*., 2018). By contrast, HopAM1’s *V*_max_ (0.0512 μM min^−1^) was low compared to that of TirS‐TIR (10 μM min^−1^), TcpC‐TIR (1.8 μM min^−1^) and SARM1 (3.57 μM min^−1^) (Essuman *et al*., 2017, 2018). Furthermore, HopAM1’s *K*_cat_ (0.56 min^−1^) is much lower than that of SARM1 (10.3 min^−1^), suggesting that HopAM1 is a slow‐turnover enzyme, consistent with the time course enzymatic activity (Fig. S2e,f) (Essuman *et al*., 2017).

### HopAM1 depletes NAD^+^ in yeast

**Fig. 3:**
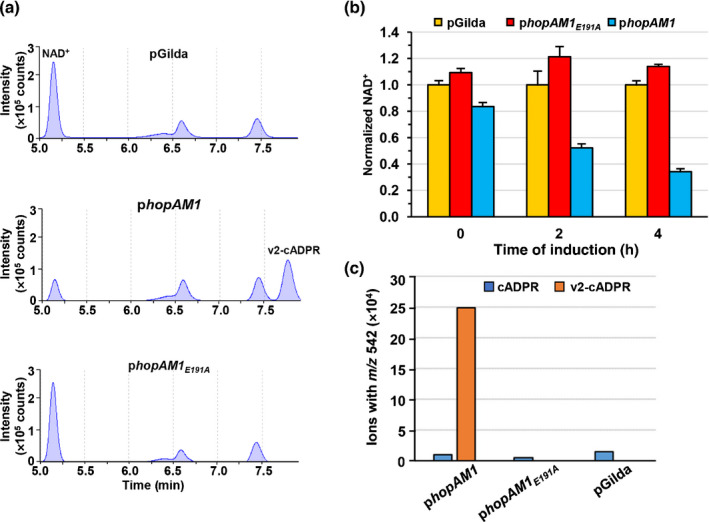
HopAM1 depletes nicotinamide adenine dinucleotide (NAD^+^) and produces v2‐cADPR in yeast. (a) A unique peak was detected with HPLC in metabolite extracts of yeast expressing HopAM1 but not HopAM1_E191A_ or pGilda vector control. (b) Decrease of NAD^+^ during the induction of HopAM1. NAD^+^ levels were normalized to pGilda empty vector control at each time point. (c) Total ion counts of cADPR and the variant cADPR derived from MALDI‐TOF (matrix‐assisted laser desorption ionization time‐of‐flight) chromatograms of the samples in (a). EGY48 yeast carrying pGilda‐*hopAM1*, pGilda‐*hopAM1_E191A_* and empty vector were induced with galactose. Metabolite samples extracted from the respective yeast culture at time 0 and 3 h after induction were subjected to HPLC and MS/MS spectrum analysis. The error bars indicate SE.

To determine if HopAM1’s TIR domain is active in yeast, we extracted metabolites from yeast cultures induced to express HopAM1, HopAM1_E191A_ or a vector control. A unique peak with a retention time of about 8 min was consistently seen in HPLC chromatograms from yeast cultures expressing HopAM1 but not HopAM1_E191A_ or the vector control at 2 h postinduction (Fig. 3a). The appearance of this unique peak was concurrent with a marked depletion of NAD^+^ (Fig. 3b), while the other peaks were relatively unchanged among all samples, consistent with the product being v2‐cADPR directly derived from endogenous NAD^+^ in yeast cells. To rule out the possibility that depletion of NAD^+^ in yeast cultures was a byproduct of cell death, we compared the amount of NAD^+^ in yeast strains expressing HopAM1 or Bax (Lacomme & Santa Cruz, 1999), a well‐known inducer of yeast cell death, and HopA1*_Psy_*_61_, an effector from *P. syringae* pv. *syringae* 61 also known to be lethal to yeast. We found the amount of NAD^+^ in all strains except HopAM1 was similar (Fig. S3a). These data show HopAM1’s NAD^+^ hydrolase activity depletes NAD^+^ to induce yeast cell death. However, it is likely that the accumulation of v2‐cADPR is also responsible for such HopAM1‐induced cell death in combination with NAD^+^ depletion or independently.

The mass of the unique metabolites was determined with LC‐MS. Metabolites with the same mass at 542 *m*/*z*, the exact mass of cADPR, were detected at retention times of 3.8 and 4.5 min, respectively (Fig. S3b). The peaks detected at 3.8 min were invariant in all three strains, apparently representing the endogenous cADPR in yeast. The peak at 4.5 min was specific to HopAM1, consistent with v2‐cADPR derived from NAD^+^ detected in the *in vitro* assay (Figs 2, S3b). Surprisingly, the HopAM1‐specific metabolite was 56‐fold more abundant than endogenous cADPR (Figs 3c, S3b). These data indicate HopAM1 hydrolyzes NAD^+^ to produce and accumulate v2‐cADPR in yeast.

### HopAM1 consumes NAD^+^ to produce v2‐cADPR in plants

**Fig. 4:**
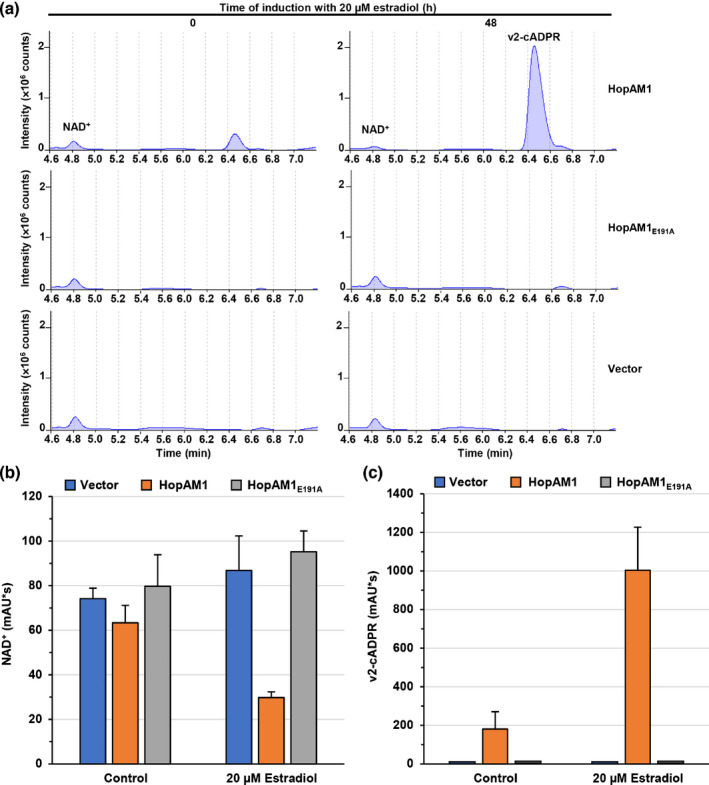
HopAM1‐dependent nicotinamide adenine dinucleotide (NAD^+^) depletion and production of variant v2‐cADPR in plants. (a) HPLC chromatograms of metabolite extracts from plants expressing HopAM1 and HopAM1_E191A_. A reduced NAD^+^ and a corresponding variant v2‐cADPR with peaks at retention times of 4.8 and 7 min in HPLC were present in samples expressing HopAM1 but not in vector control or HopAM1_E191A_. Metabolites from control plant leaves or those induced with 20 µM estradiol for 48 h were analyzed with HPLC. (b) Quantification of NAD^+^ and (c) variant v2‐cADPR production shown in (a). *Nicotiana benthamiana* leaves were infiltrated with *Agrobacterium* strains harboring the vector control, p*hopAM1* and p*hopAM1_E191A_* and after 24 h were sprayed with 20 µM estradiol. Metabolites were extracted from induced plants and noninduced controls 48 h after estradiol application. The error bars indicate SE.

To examine if HopAM1 is also active in plants, we transiently expressed HopAM1 and HopAM1_E191A_, both fused with an HA epitope tag and driven with estradiol inducible promoter, in *N. benthamiana* and subsequently extracted metabolites 48 h after estradiol induction. As in yeast, HPLC revealed that metabolic extracts from HopAM1‐expressing samples contained a unique peak with a retention time of the product v2‐cADPR concurrent with NAD^+^ depletion (Figs 2, 4a–c). In contrast, no metabolic changes were observed in samples expressing the TIR catalytic null mutant HopAM1_E191A_ or the vector control (Fig. 4a). Overexpression of HopAM1 but not HopAM1_E191A_ via estradiol induction further depleted endogenous NAD^+^ in the plants (Fig. 4b), validating that HopAM1 is active in consuming NAD^+^ *in planta*. Only estradiol‐induced HopAM1 overexpressions showed cell death, suggesting that that HopAM1‐associated cell death in *N. benthamiana* may be the result of either NAD^+^ depletion or the accumulation of v2‐cADPR or a combination of both via HopAM1’s endogenous activity (Figs 1d,e, 4b,c).

### T3SS delivered HopAM1 is active in Arabidopsis plants

**Fig. 5:**
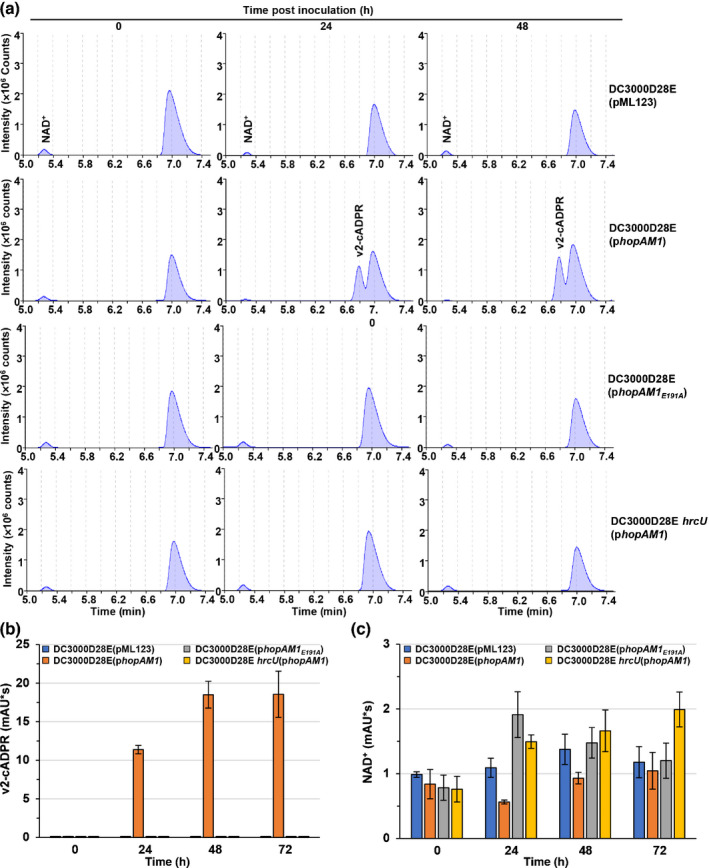
Type III secretion system (T3SS)‐delivered HopAM1 possesses *in vivo* activity in Arabidopsis. (a) HPLC analysis of metabolites in Arabidopsis Col‐0 leaves infected with DC3000D28E expressing HopAM1, HopAM1_E191A_, empty vector control or T3SS‐defective mutant DC3000D28E *hrcU*. (b) Quantification of v2‐cADPR *in planta* production and (c) nicotinamide adenine dinucleotide (NAD^+^) of the samples infected with the strains in (a). Values are means ± SE. The amount of v2‐cADPR is elevated by HopAM1 while NAD^+^ levels are not significantly altered in plants.

To determine whether HopAM1 activity is detectable in plant cells after type III secreted delivery by bacteria, *Pto* DC3000D28E was used to express HopAM1 and HopAM1_E191A_ *in trans* via a broad host vector. *Pto* DC3000D28E is a polyeffector mutant of *Pto* DC3000 lacking 28 key effector genes, including both alleles of *hopAM1*, and is nearly nonpathogenic in Arabidopsis and *N. benthamiana* (Cunnac *et al*., 2011; Velásquez *et al*., 2017). HopAM1 triggers a hypersensitive‐like cell death response in resistant Arabidopsis accessions Xan‐2 and Xan‐5 (Velásquez *et al*., 2017). We then assessed metabolic changes over time in both Arabidopsis ecotypes Col‐0 and Xan‐2 infiltrated with *Pto* DC3000D28E(pML123‐*hopAM1*), *Pto* DC3000D28E(pML123‐*hopAM1_E191A_*), *Pto* DC3000D28E(pML123), as well as the T3SS defective strain *Pto* DC3000D28E *hrcU*(pML123‐*hopAM1*). Expression of HA‐tagged HopAM1 and its mutants in DC3000D28E strains was detected with immunoblots and appeared to be stable (Fig. S6a). v2‐cADPR was detected within 24 h postinoculation with *Pto* DC3000D28E(pML123‐*hopAM1*) and the amount appeared to increase over the course of infection (Figs 5a,b, S4a). Nam was not distinguishable due to coelution with a large peak of another metabolite in common for all Arabidopsis samples. Neither *Pto* DC3000D28E(pML123‐*hopAM1_E191A_*), the vector control, nor type III defective strain *Pto* DC3000D28E *hrcU*(pML123‐*hopAM1*) were able to produce v2‐cADPR in plants (Fig. 5a). Interestingly, despite the clear accumulation of v2‐cADPR, no significant changes were detected in the amount of NAD^+^ among plants infected with different strains, suggesting that DC3000 T3SS‐injected HopAM1 is sufficient to accumulate v2‐cADPR but insufficient to overcome NAD^+^ homeostasis (Figs 5a–c, S4a,b).

To rule out the possibility that other effectors in *Pto* DC3000 also possess the same NAD^+^ hydrolase activity, we analyzed metabolites in Arabidopsis plants infected with DC3000 mutants lacking one or both *hopAM1‐1* and *hopAM1‐2* alleles. The metabolic profile of *Pto* DC3000‐infected Arabidopsis Col‐0 was similar to that of *Pto* DC3000D28E(pML123‐*hopAM1*), whereas reduced amounts of v2‐cADPR were detected in leaves infected with the *ΔhopAM1‐1* and *ΔhopAM1‐2* single mutants (Figs 5a,b, S5a,b). No v2‐cADPR was detected in leaves infected with the *ΔhopAM1‐1 ΔhopAM1‐2* (*ΔhopAM1‐1/1‐2*) double mutant lacking both *hopAM1* alleles, but complementation with pML123‐*hopAM1* was sufficient to restore v2‐cADPR production to wild‐type levels (Fig. S5a,b). Consistent with *Pto* DC3000D28E strains, the amount of NAD^+^ was not significantly altered during infection (Figs 5c, S5c). Together, these data show HopAM1 is the sole effector in *Pto* DC3000 and each allele encodes an active enzyme that displays NAD^+^ hydrolase activity in plants after delivery via the native *Pto* DC3000 T3SS.

### HopAM1’s putative catalytic residue is required for ETI‐like responses in resistant Arabidopsis plants

**Fig. 6:**
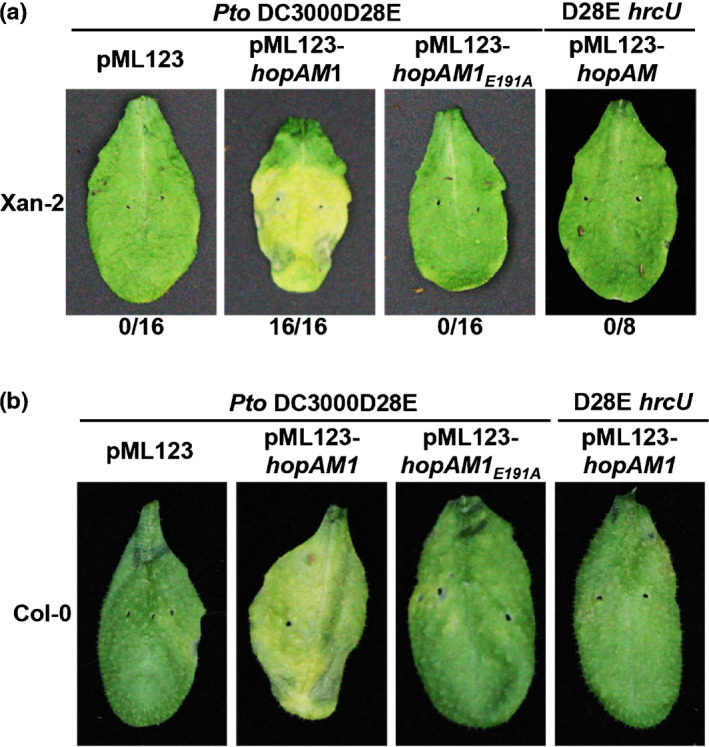
HopAM1‐induced responses in different Arabidopsis accessions. (a) Hypersensitive response‐like response in resistant Arabidopsis accession Xan‐2. (b) Virulence response in susceptible Arabidopsis Col‐0. Development of symptom‐like chlorosis was seen only in Col‐0 infiltrated with *Pto* DC3000D28E (pML123‐*hopAM1*), not vector control *Pto* DC3000D28E(pML123), *Pto* DC3000D28E(p*hopAM1_E191A_*) or *Pto* DC3000D28E *hrcU*(pML123‐*hopAM1*). *Pto* DC3000D28E or *Pto* DC3000D28E *hrcU* strains carrying the respective plasmids were infiltrated into Arabidopsis leaves at a cell density of 1 × 10^8^ cells ml^−1^. Images were taken 4 d after inoculation.

To examine whether HopAM1’s putative catalytic residue is required for ETI‐like responses, Xan‐2 and Xan‐5 were inoculated with *Pto* DC3000D28E strains expressing HopAM1 or the catalytic null mutant HopAM1_E191A_, as well as T3SS‐defective strain DC3000D28E *hrcU*(pML123‐*hopAM1*). When infiltrated with *Pto* DC3000D28E(p*hopAM1*) at 1 × 10^8^ cells ml^−1^, leaves of both Xan‐2 and Xan‐5 displayed an ‘ETI‐like’ cell death hallmarked by severe chlorosis, consistent with the previous observation (Velásquez *et al*., 2017) (Figs 6a, S6b). By contrast, no chlorosis developed in leaves infiltrated with DC3000D28E(p*hopAM1_E191A_*), DC3000D28E *hrcU*(pML123‐*hopAM1*) or the vector control (Figs 6a, S6b). When the assay was repeated in susceptible Arabidopsis Col‐0 plants, a symptom‐like mild chlorosis or yellowing was observed in plants infiltrated with *Pto* DC3000D28E(p*hopAM1*) often present with less severity than the chlorosis in Xan‐2 and Xan‐5, suggesting a virulent response in the susceptible Arabidopsis host (Figs 6a,b, S6b). Interestingly, ETI‐like responses were also abolished in Xan‐5 infiltrated with DC3000D28E(p*hopAM1_F177A_*) (Fig. S6b), suggesting that other conserved residues in the putative TIR domain may also affect HopAM1’s NAD^+^ hydrolase activity. It appears HopAM1’s activity has a prominent effect on symptom development. HopAM1’s putative catalytic residue is required for both ETI‐like responses in Xan‐2 and Xan‐5 plants and a symptom‐like chlorosis response in Col‐0, suggesting that there is a spectrum of genotype‐dependent responses to HopAM1’s NAD^+^ hydrolysis and v2‐cADPR production *in planta* (Figs 5, 6, S4, S5).

### *Pto* DC3000 HopAM1 virulence is dependent on its putative catalytic residue

**Fig. 7:**
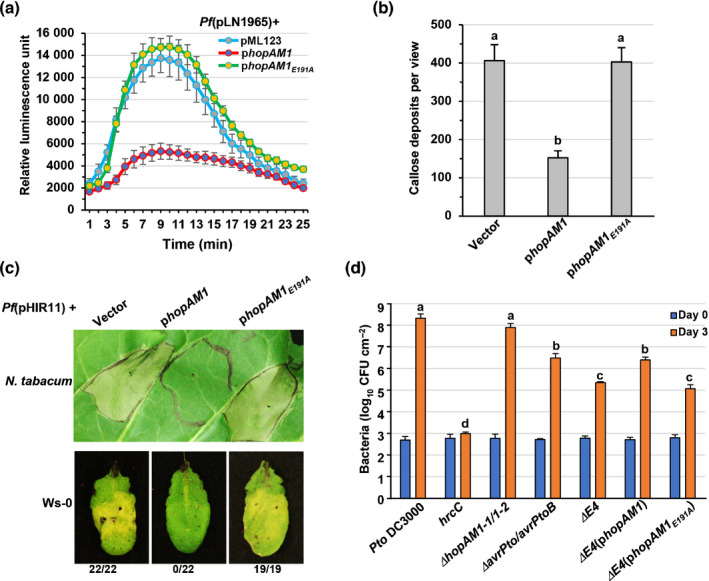
HopAM1 requires putative catalytic glutamic acid residue E191 to suppress pattern‐triggered immunity and effector‐triggered immunity and to contribute to virulence. (a) Reactive oxygen species (ROS) assays with *Pf*(*pLN1965*) strains carrying pML123 (Vector), pML123‐*hopAM1* and pML123‐*hopAM1_E191A_*. *Pf*(*pLN1965*) strains were infiltrated into Arabidopsis Col‐0 leaves and ROS production was determined as relative luminescence. (b) Callose deposition assays of *Pf*(*pLN1965*) strains used in (a). (c) Hypersensitive response (HR) suppression assays in *Nicotiana tabacum* cv Xanthi (upper panel) and Arabidopsis Ws‐0 (lower panel) with *Pf*(pHIR11) strains carrying pML123 (vector), pML123‐*hopAM1* or pML123‐*hopAM1_E191A_*. (d) *In planta* bacterial growth in Arabidopsis Col‐0. Pathogenicity assays were conducted with *Pto* DC3000, *hrcC*, *a* type III defective mutant, a double mutant *ΔhopAM1‐1 ΔhopAM1‐2* (*ΔhopAM1‐1/1‐2*), a double mutant *ΔavrPto ΔavrPtoB* and a quadruple mutant *ΔavrPto ΔavrPtoB ΔhopAM1‐1 ΔhopAM1‐2* (*ΔE4*), and the complementing strains expressing HopAM1 and HopAM1_E191A_. *Pf*(pHIR11) is a *Pseudomonas fluorescens* strain carrying a cosmid pHIR11 that encodes a full functional type III secretion system and effector HopA1 derived from *Pseudomonas syringae* pv. *syringae* 61, which enables delivery of HopA1 to trigger an HR in *N. tabacum* cv Xanthi and Arabidopsis Ws‐0 in a manner dependent on RPS6. Arabidopsis leaves were spray‐inoculated with the indicated strains. Bacterial growth in leaf tissue was quantified 0 and 3 d after inoculation. Bars denote SE. Letters denote statistical significance (*P* < 0.05). The bacterial growth assays with Col‐0 were repeated three times with similar results.

HopAM1 suppresses both plant PTI and ETI (Jamir *et al*., 2004; Goel *et al*., 2008; Guo *et al*., 2009). To examine if HopAM1 PTI suppression is dependent on its TIR domain, we utilized the nonpathogenic *Pseudomonas fluorescens* (*Pf*)(pLN1965) strain, which expresses a functional T3SS and delivers heterologous type III effectors effectively for immunity suppression assays (Guo *et al*., 2009). Arabidopsis Col‐0 leaves were infiltrated with *Pf*(pLN1965) strains expressing HopAM1, putative catalytic mutant HopAM1_E191A_ and a vector control and assayed for ROS production and callose deposition. *Pf*(pLN1965) expressing HopAM1 suppressed ROS production and callose deposition, while HopAM1_E191A_ and the vector failed to suppress either response (Fig. 7a,b). HopAM1 was previously shown to suppress ETI in *N. tabacum* and Arabidopsis Ws‐0 induced by *Pf*(pHIR11), which encodes a functional T3SS and HopA1*_Psy_*_61_, an effector that triggers an ETI dependent on the TIR‐NLR RPS6 in Arabidopsis (Jamir *et al*., 2004; Kim *et al*., 2009). HopAM1 could suppress *Pf*(pHIR11)‐dependent ETI while HopAM1_E191A_ and the vector control could not (Fig. 7c). These results clearly demonstrate that a putative catalytic site within the TIR domain is required for HopAM1’s suppression of plant immune responses.

We further determined how HopAM1 contributes to *Pto* DC3000 virulence in Arabidopsis Col‐0 and tomato cv Money Maker plants. In assays with *hopAM1* single mutants, neither *ΔhopAM1‐1* nor *ΔhopAM1‐2* was reduced in bacterial growth relative to the wild‐type, consistent with a previous report (Boch *et al*., 2002). The *ΔhopAM1‐1/1‐2* double mutant displayed a slight but insignificant reduction in bacterial growth in Col‐0 and tomato plants (Figs 7d, S7). Due to redundancy of effectors in DC3000, single effector mutants often produce only subtle growth defects while polyeffector mutants more clearly demonstrate variations in virulence (Cunnac *et al*., 2011). To examine HopAM1’s contribution to *Pto* DC3000 virulence, HopAM1 was deleted in tandem with *avrPto* and *avrPtoB*, two well‐known key effectors in *Pto* DC3000. As expected, the *ΔavrPto ΔavrPtoB* double mutant displayed reduced growth in Col‐0 and tomato (Figs 7d, S7) (Lin & Martin, 2005). A quadruple effector mutant *ΔhopAM1‐1 ΔhopAM1‐2 ΔavrPto ΔavrPtoB* (*ΔE4*) was generated by further deletion of both *hopAM1‐1* and *hopAM1‐2* from the *ΔavrPto ΔavrPtoB* double mutant and assessed for its *in planta* bacterial growth. *ΔE4* was further reduced relative to *ΔavrPto ΔavrPtoB*, and complementation with HopAM1 but not HopAM1_E191A_ was able to restore the growth of *ΔE4* to the level of *ΔavrPto ΔavrPtoB* (Figs 7d, S7). Bacterial growth assays indicate HopAM1 contributes to DC3000 virulence in plants in a manner dependent on the putative catalytic residue within its TIR domain concurrent with production of v2‐cADPR.

## Discussion

We report that the *Pto* DC3000 effector HopAM1 contains a noncanonical TIR domain and possesses enzymatic activity that hydrolyzes NAD^+^ *in vitro* and produces nicotinamide and the novel metabolite v2‐cADPR, likely to be a unique cADPR isomer distinct from cADPR and v‐cADPR (Fig. 2). The existence of two cADPR variants suggests a family of alternative cyclic linkages for cADPR. The *in vivo* NAD^+^ hydrolase activity and production of v2‐cADPR is evidence that overexpression of HopAM1 in yeast and plants results in a drastic decrease of endogenous NAD^+^ (Figs 3, 4). Moreover, HopAM1’s enzymatic activity is detectable *in planta* after native T3SS delivery and is required for HopAM1’s suppression of plant immunity and contribution to *Pto* virulence (Figs 5, 6, 7). Although the majority of *P. syringae* type III effectors appear to suppress plant immunity and collectively contribute to virulence, the biochemical activity of most effectors is still elusive (Guo *et al*., 2009; Block & Alfano, 2011; Büttner, 2016). Most enzymatically active effectors of plant pathogens biochemically modify immunity‐associated host proteins to alter or suppress immunity and promote virulence (Block & Alfano, 2011). Only a few effectors have been demonstrated to interfere with plant metabolism or directly target essential metabolites of host plants (Poueymiro *et al*., 2014; Shidore *et al*., 2017). The study highlights a clearly novel biochemical activity for a phytopathogenic effector that hydrolyzes the essential metabolite NAD^+^ to generate a novel metabolite that probably increases virulence in host plants.

In plants, the NAD^+^ hydrolase activity of TIR domain‐containing NLRs during activation of immunity was recently revealed to be essential to the perception of cognate effectors and ETI (Horsefield *et al*., 2019; Wan *et al*., 2019; Ma *et al*., 2020; Martin *et al*., 2020). Though both HopAM1 and a subset of plant NLRs contain TIR domains, HopAM1 is distinguished by several key differences. While plant NLR NAD^+^ hydrolase activity activates immunity, HopAM1’s NAD^+^ hydrolase activity suppresses it. HopAM1 is active in different subcellular compartments than plant NLRs: HopAM1 localizes to the cytoplasm and probably acts in the cytosol (Fig. S8), whereas plant NLRs are plasma membrane‐bound (Dangl *et al*., 2013; Choi *et al*., 2017). More importantly, despite the same substrate NAD^+^, HopAM1’s product is different. HopAM1‐induced cell death is not EDS1‐dependent, suggesting it does not induce typical ETI‐associated cell death (Choi *et al*., 2017). It is most likely that TIR domain effectors such as HopAM1 evolved specific biochemical activity to generate a novel metabolite to subvert host immunity and promote virulence.

Toll/interleukin‐1 receptor domain‐containing proteins belong to a newly emerging class of virulence factors that are widely distributed among various animal bacterial pathogens, though they are also commonly seen in nonpathogenic organisms (Spear *et al*., 2009; Cirl & Miethke, 2010). Despite controversy, much attention has been given to the theory that bacteria have adapted TIR domain proteins to interfere with the host immune system via protein–protein interactions and molecular mimicry to promote virulence (Spear *et al*., 2009). However, recent findings that both eukaryotic and bacterial TIR domains belong to a large ancient family of NAD^+^ hydrolases have led to a better understanding of TIR domain‐containing virulence and effector proteins (Essuman *et al*., 2018; Essuman, 2020). Recently, the TIR domain‐containing type IV effectors BtpA and BtpB from *B. abortus* were found to interfere with NAD^+^ metabolism in human cells, suggesting that TIR domain enzymatic activity is important for virulence in animals (Coronas‐Serna *et al*., 2020). HopAM1 is an unprecedented phytopathogenic effector injected by the *Pto* T3SS that exerts NAD^+^ hydrolase activity *in planta* to promote virulence, reinforcing TIR domain‐associated NAD^+^ hydrolase activity as a component of pathogen virulence in diverse eukaryotic hosts.

ABA is a signaling target of plant bacterial pathogens. Elevated levels of ABA or increased sensitivity to ABA appear to be correlated with *P. syringae* virulence and enhancement of bacterial growth (Cao *et al*., 2011). *Pseudomonas syringae* type III effectors manipulate the ABA pathway or undermine the ABA network (de Torres‐Zabala *et al*., 2007; García‐Andrade *et al*., 2020). AvrPtoB promotes ABA accumulation for *P. syringae* virulence. Unlike AvrPtoB, HopAM1 and HopF2 act similarly to enhance ABA sensitivity and suppress immune responses for *P. syringae* virulence, but the underlying biochemical mechanism is not known (de Torres‐Zabala *et al*., 2007; Goel *et al*., 2008; Cao *et al*., 2019). However, recent studies have revealed an unexpected link between NAD^+^ and the ABA response pathway with NAD^+^ regulation probably upstream of ABA signaling (Feitosa‐Araujo *et al*., 2020; Hong *et al*., 2020). While endogenous NAD^+^ levels in plant leaves are depleted as a consequence of HopAM1 overexpression via *Agrobacterium*‐mediated transformation, NAD^+^ homeostasis is not changed significantly by T3SS‐delivered HopAM1. By contrast, HopAM1 mediates substantial accumulation of v2‐cADPR following both heterologous overexpression and native delivery. Thus, it is possible that previously observed effects of HopAM1 on ABA responses are indirect (Lee, 1994; Goel *et al*., 2008). Alternatively, given that cADPR acts as second messenger of ABA and v2‐cADPR is an isomer of cADPR, it is plausible that HopAM1‐mediated v2‐cADPR affects ABA responses by interfering with cADPR signaling (Wu *et al*., 1997).

HopAM1 induces HR‐like cell death in some plants while producing mild chlorosis in others. HopAM1 was originally identified in *P. syringae* pv. *pisi* as a plasmid‐borne avirulence gene *avrPpiB* based on the HR response in pea cultivars with specific R3 resistance locus (Cournoyer *et al*., 1995). HopAM1 induces cell death in *Nicotiana* species, but it is not clear if this recognition is specific to an R protein. HopAM1‐associated cell death is dependent on HopAM1’s catalytic activity and is observed both in experiments showing NAD^+^ depletion and those without significant NAD^+^ depletion. In mammalian neurons, SARM1‐mediated hydrolysis of NAD^+^ is the mechanism of execution of axon degeneration (Gerdts *et al*., 2016; Essuman *et al*., 2017). Activation of SARM1 results in NAD^+^ depletion and marked production of cADPR, but modulation of cADPR has no effect on axon degeneration (Sasaki *et al*., 2020). This model suggests that depletion of endogenous NAD^+^ may be one cause of HopAM1‐induced cell death seen in plant and yeast over‐expressions. Another cause of HopAM1‐induced cell death may be accumulation of v2‐cADPR. It is not clear whether HopAM1‐dependent cell death or severe chlorosis seen in Arabidopsis genotypes Xan‐2 and Xan‐5 is the same as cell death observed in genotype Bur‐0 and lesions seen in *N. benthamiana* (Cunnac *et al*., 2011; Iakovidis *et al*., 2016; Velásquez *et al*., 2017). Resistance of Xan‐2 and Xan‐5 to *hopAM1*‐carrying strains is atypical: instead of qualitative NLR (R)‐mediated resistance, two major quantitative trait loci are associated with HopAM1‐induced cell death and chlorosis (Iakovidis *et al*., 2016). It is possible that Xan‐2 and Xan‐5 do not exhibit canonical resistance but rather quantitative traits conferring sensitivity to HopAM1‐produced v2‐cADPR. HopAM1‐associated chlorosis in Col‐0 is less severe and resembles that seen in Ws‐0 upon bacterial expression of HopAM1 *in trans* or transgenic expression *in planta* (Goel *et al*., 2008). Unlike Xan‐2 and Xan‐5, both Col‐0 and Ws‐0 are susceptible to *Pto* DC3000, which suggests chlorosis results from virulence activity rather than an immune response (Goel *et al*., 2008). We confirmed that HopAM1‐associated chlorosis is reliant on HopAM1’s activity (Fig. 6), perhaps as a direct consequence of accumulation of the product v2‐cADPR. Interestingly, *hopAM1* is absent in group II *P. syringae* strains that contain toxin (syringomycin, syringopeptin and syringolin) biosynthesis pathways (Baltrus *et al*., 2011). Nontoxin‐producing *P. syringae* strains may have acquired effector genes such as *hopAM1* to take advantage of existing metabolites in hosts to produce *de novo* small molecules for symptom development and virulence during infection. The small molecule effector victorin of the necrotrophic fungus *Cochliobolus victoriae* behaves differently in the presence or absence of an NLR to induce host susceptibility (Lorang *et al*., 2012). Similarly, it is plausible that differences in sensitivity to v2‐cADPR lead to differential responses in the context of genotypes of different susceptibility. The identification of more effector‐mediated small molecules and determination of their role in pathogenesis deserve further investigation.

When delivered via DC3000, HopAM1 does not measurably deplete NAD^+^ but still results in v2‐cADPR accumulation. This suggests that, although NAD^+^ is an essential plant metabolite and coenzyme with involvement in immunity, HopAM1’s contribution to virulence may not be entirely due to depletion of NAD^+^ but instead accumulation of v2‐cADPR (Fig. 5). The chemical structures of v‐cADPR and v2‐cADPR remain to be resolved. The specific function of v2‐cADPR and its relationship to cADPR and v‐cADPR are still unknown in the context of either eukaryotic NLR‐associated immune activity or prokaryotic HopAM1‐associated virulence activity. However, the apparent similarity of v2‐cADPR to cADPR and v‐cADPR suggests that this novel isoform may interfere with cADPR and v‐cADPR signaling downstream of plant NLRs in order to suppress immunity.

## Author contributions

MG conceived experiments. SE carried out most experiments and TS conducted HPLC analysis. MAZ and ND conducted kinetics experiments of HopAM1 and AbTIR. PK performed immunoblots to detect expression in yeast. SM conducted immunoblots to detect expression in bacterial strains. AD and JM provided key materials and insights for NAD^+^ hydrolase experiments. JRA provided intellectual insights. TEC oversaw the experiments. SE and MG contributed to manuscript writing.

## Supporting information


**Fig. S1** HopAM1 contains a putative Toll/interleukin‐1 receptor domain.
**Fig. S2** HopAM1 hydrolyzes nicotinamide adenine dinucleotide *in vitro*.
**Fig. S3** HopAM1’s nicotinamide adenine dinucleotide (NAD^+^) hydrolysis activity is associated with NAD^+^ depletion in yeast.
**Fig. S4** HopAM1‐mediated metabolites in Arabidopsis Xan‐2.
**Fig. S5** HopAM1 in *Pto* DC3000 is responsible for production of v2‐cADPR in Arabidopsis.
**Fig. S6** HopAM1’s effector‐triggered immunity‐like response in Arabidopsis Xan‐5 is dependent on its conserved residues in the Toll/interleukin‐1 receptor domain.
**Fig. S7** HopAM1 contributes to *Pto* DC3000 virulence in tomato plants in a manner dependent on its putative catalytic residue.
**Fig. S8** Mutation of conserved residues in HopAM1’s Toll/interleukin‐1 receptor domain do not affect its subcellular localization.
**Methods S1** Supporting Information for the Materials and Methods.
**Table S1** Strains and plasmids used in this study.
Please note: Wiley Blackwell are not responsible for the content or functionality of any Supporting Information supplied by the authors. Any queries (other than missing material) should be directed to the *New Phytologist* Central Office.

## Data Availability

All vectors and strains are available by request to Ming Guo at mguo2@unl.edu.
