# Supplementary material for: A phytobacterial TIR domain effector manipulates NAD+ to promote virulence
Source: New Phytol. 2021 Nov 5;233(2):890–904. doi: 10.1111/nph.17805 (PMC9298051; doi:10.1111/nph.17805)
Supplement: Supplementary file 1 — Fig. S1 HopAM1 contains a putative Toll/interleukin‐1 receptor domain. Fig. S2 HopAM1 hydrolyzes nicotinamide adenine dinucleotide in vitro. Fig. S3 HopAM1’s nicotinamide adenine dinucleotide (NAD+) hydrolysis activity is associated with NAD+ depletion in yeast. Fig. S4 HopAM1‐mediated metabolites in Arabidopsis Xan‐2. Fig. S5 HopAM1 in Pto DC3000 is responsible for production of v2‐cADPR in Arabidopsis. Fig. S6 HopAM1’s effector‐triggered immunity‐like response in Arabidopsis Xan‐5 is dependent on its conserved residues in the Toll/interleukin‐1 receptor domain. Fig. S7 HopAM1 contributes to Pto DC3000 virulence in tomato plants in a manner dependent on its putative catalytic residue. Fig. S8 Mutation of conserved residues in HopAM1’s Toll/interleukin‐1 receptor domain do not affect its subcellular localization. Methods S1 Supporting Information for the Materials and Methods. Table S1 Strains and plasmids used in this study. Please note: Wiley Blackwell are not responsible for the content or functionality of any Supporting Information supplied by the authors. Any queries (other than missing material) should be directed to the New Phytologist Central Office. [file NPH-233-890-s001.pdf]

Supporting Information

Article title: **A phytobacterial TIR domain effector manipulates NAD^+ to promote virulence**

Authors: Samuel Eastman, Thomas Smith, Mark A. Zaydman, Panya Kim, Samuel Martinez, Neha Damaraju, Aaron DiAntonio, Jeffrey Milbrandt, Thomas E. Clemente, James R. Alfano[†], Ming Guo: 15 September 2021

The following Supporting Information is available for this article:

[†]Deceased.

Fig. S1 HopAM1 contains a putative TIR domain

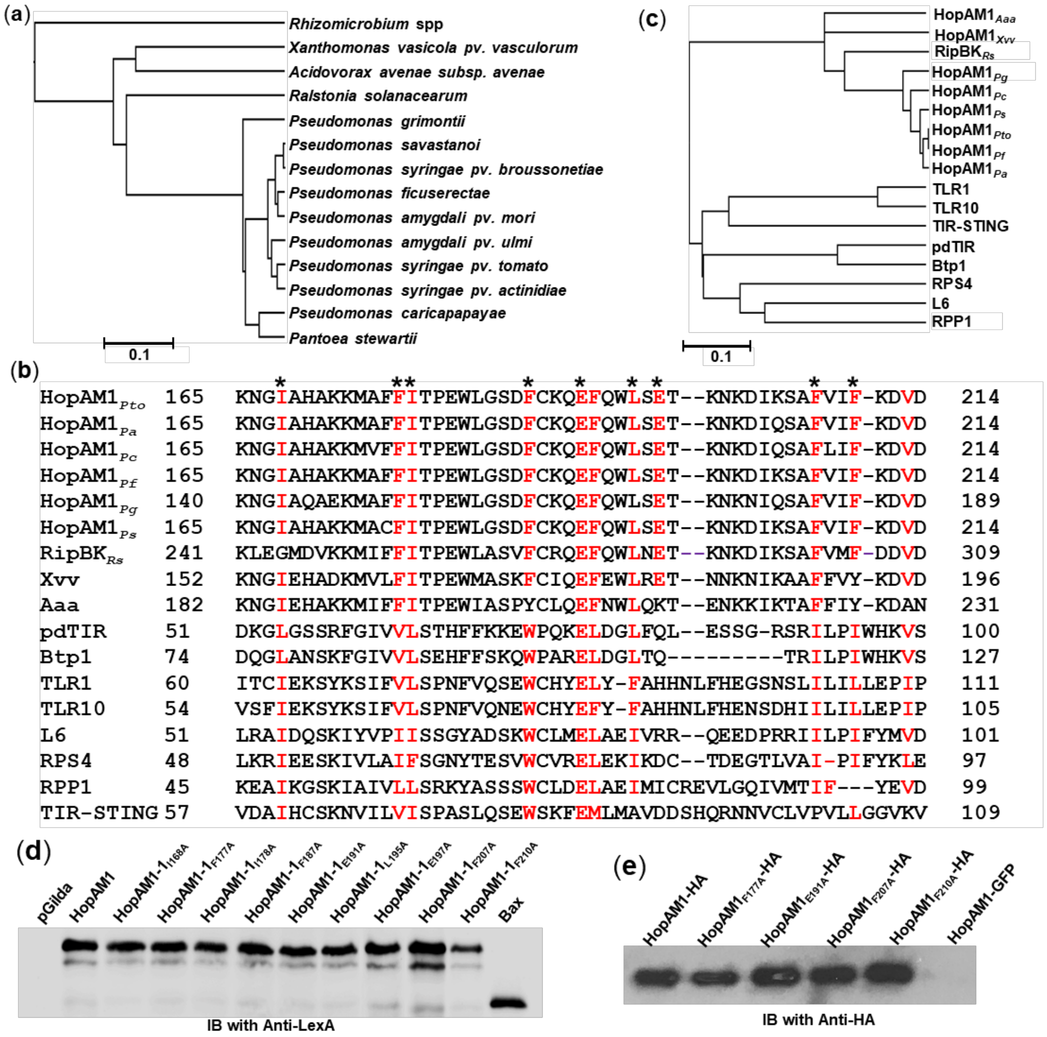

Fig. S1. HopAM1 contains a putative TIR domain. (**a**) A phylogenetic tree of HopAM1 homologs that are moderately distributed in phytobacterial pathogens. (**b**) Alignment of HopAM1's putative TIR-domain with known TIR-domains. HopAM1$_{Pto}$, *P. syringae* pv. tomato DC3000; HopAM1$_{Pa}$ (WP_005730427.1), (*P. amygdali*); HopAM1$_{Pc}$ (WP_122341221.1), *P. caricapapayae*; HopAM1$_{Pf}$ (WP_054995632.1), *P. ficuserectae*; HopAM1$_{Pg}$ (WP_090403225.1), *P. grimontii*; HopAM1$_{Ps}$ (RMS28091.1), *Pseudomonas savastanoi*; RipBKRs (NKA56149.1), *Ralstonia solanacearum*; Xvv (KFA22784.1), *Xanthomonas vasicola* pv. *vasculorum*; Aaa (CP028289.1), *Acidovorax avenae* subsp. *avenae* strain AA99_2; pdTIR, (*Paracoccus denitrificans*); Btp1, *Brucella* TIR protein 1 (*Brucella abortus*)；TLR1 Toll-like receptor 1; TLR10，Toll-like receptor 10; L6, disease resistance protein of flax (*Linum usitatissimum*); RPS4, Arabidopsis resistance protein; R4, Arabidopsis disease resistance protein RPP1-like (recognition of *peronospora parasitica* 1) (ACJ64858.1); TIR-STING, metazoan TIR-STING (stimulator of interferon genes protein) receptor (*Crassostrea gigas*). Conserved and similar amino acid residues were shown in red. Asterisks (*) on the top of the alignment indicate the residues that were site-directed mutated and examined in yeast cell death assay and a subset was tested in HR assay in plants shown in Fig. 1. (**c**) A phylogenetic tree of the TIR domain sequences of the proteins shown in (**a**). (**d**) Immunoblots show expression of HopAM1 and its mutants examined in yeast strains in Fig. **1c** and in plants in Fig. **1d, e**.

Fig. S2 HopAM1 hydrolyzes NAD$^+$ *in vitro*

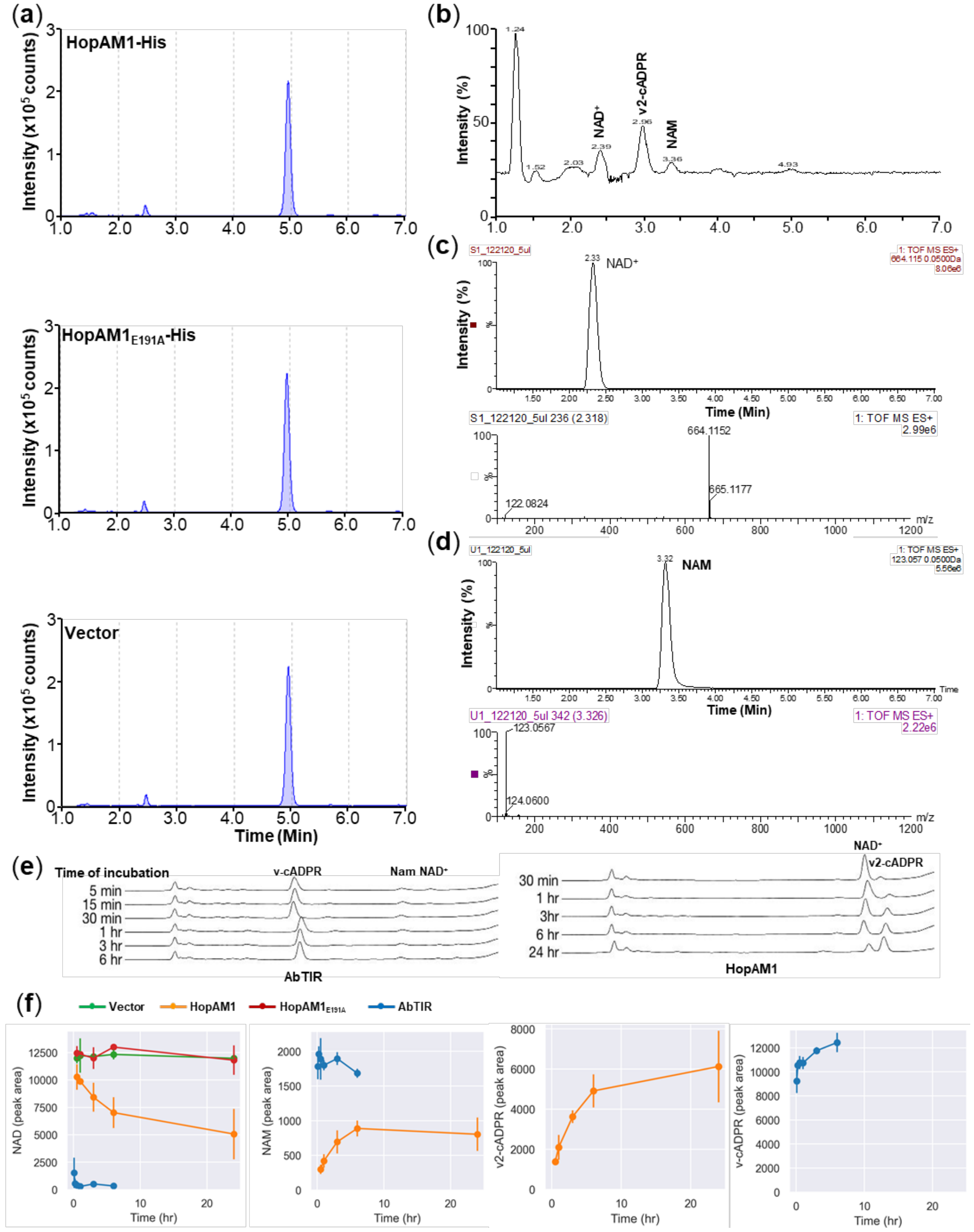

Fig. S2 HopAM1 hydrolyze NAD$^+$ *in vitro*. (**a**) Time 0 of *in vitro* assays with purified HopAM1-His and HopAM1$_{E191A}$-His proteins, and mock preparation of vector control. (**b**) HPLC chromatograms of *in vitro* reaction with HopAM1 protein. **(c)** and **(d)** Mass spectrum of NAD$^+$, and NAM in an *in vitro* reaction, respectively. (**e**) Chromatographs of time course enzymatic reactions with purified AbTIR and HopAM1. (**f**) Time course enzymatic activity showing NAD$^+$ hydrolysis by HopAM1, HopAM1$_{E191A}$, and AbTIR. Bars denote standard error (SE).

Fig. S3 HopAM1's NAD$^+$ hydrolysis activity is associated with NAD$^+$ depletion in yeast

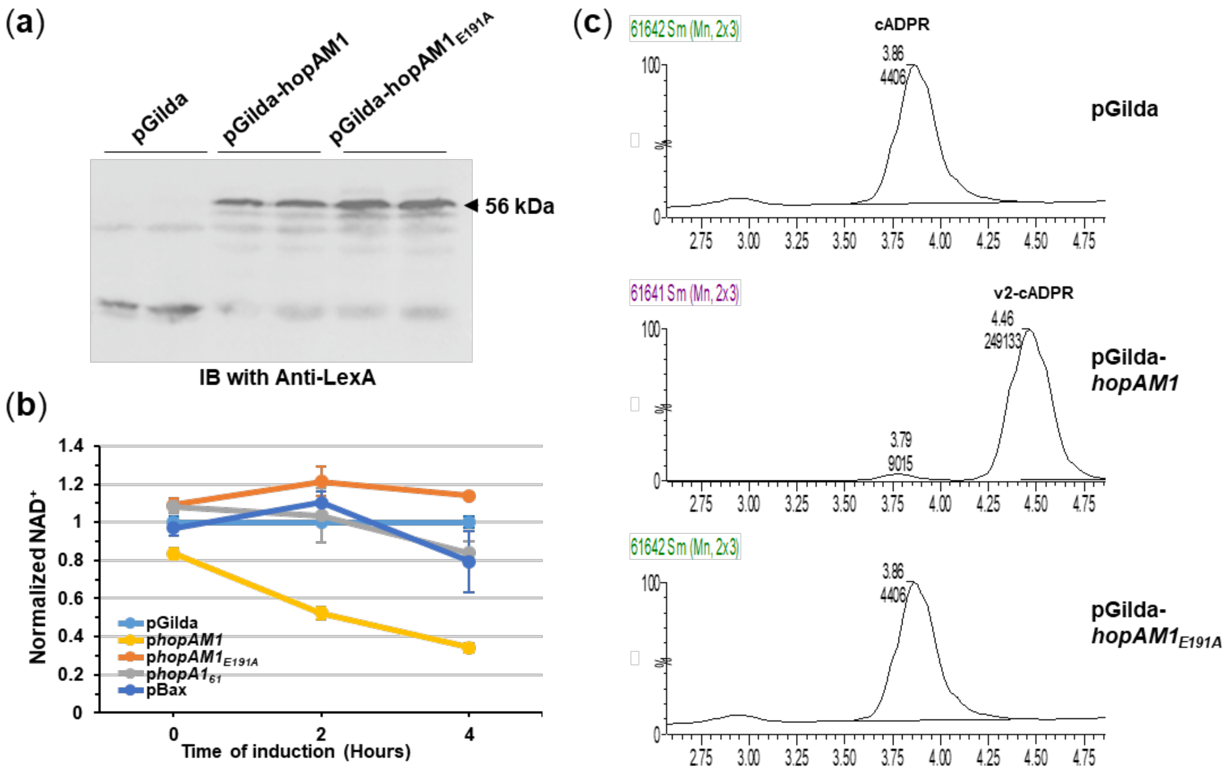

Fig. S3 HopAM1's NAD$^+$ hydrolyzing activity is associated with NAD$^+$ depletion in yeast. (**a**) Immunoblots detecting expression of HopAM1 and HopAM1$_{E191A}$ galactose-induced yeast cultures. (**b**) Normalized NAD$^+$ levels of the yeast strains upon the expression of HopAM1, HopAM1$_{E191A}$, HopA1$_{Psy61}$, Bax and a vector control. Expression of HopAM1, HopA1$_{Psy61}$, and Bax all have result in growth defect in yeast. Bars indicate standard error. (**c**) The variant v2-cADPR peak was eluted around 4.5 minutes, while the endogenous canonical cADPR at around 3.8 min with both identified to have the same 542 m/z as cADPR. The results here indicated that decrease of NAD$^+$ is specifically associated with HopAM1 and dependent on its putative catalytic site of TIR domain and independent of yeast cell death triggered by either HopAM1 or the other PCD inducers in yeast. HopA1$_{Psy61}$ is a type III effector from *P. syringae* pv. syringae 61 and Bax is well-known pro-apoptosis protein, both are lethal when expressed in yeast.

Fig. S4 HopAM1-mediated metabolites in Arabidopsis Xan-2

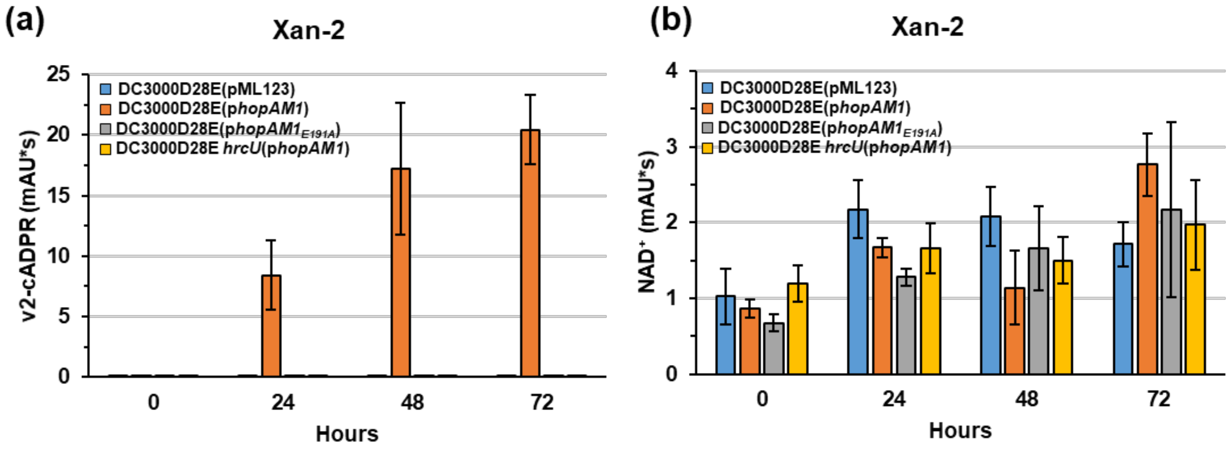

Fig. S4 HopAM1-mediated metabolites in Arabidopsis Xan-2. (**a**) v2-cADPR and (**b**) NAD^+ in Xan-2 plants infected with *Pto* DC3000D28E strains. *Pto* DC3000D28E strains carrying respective plasmids were infiltrated into Arabidopsis Xan-2 leaves at cell density of 2 x 10^8 cells ml^{-1}. Bars denote standard error.

Fig. S5 HopAM1 in *Pto* DC3000 is responsible to produce v2-cADPR in Arabidopsis

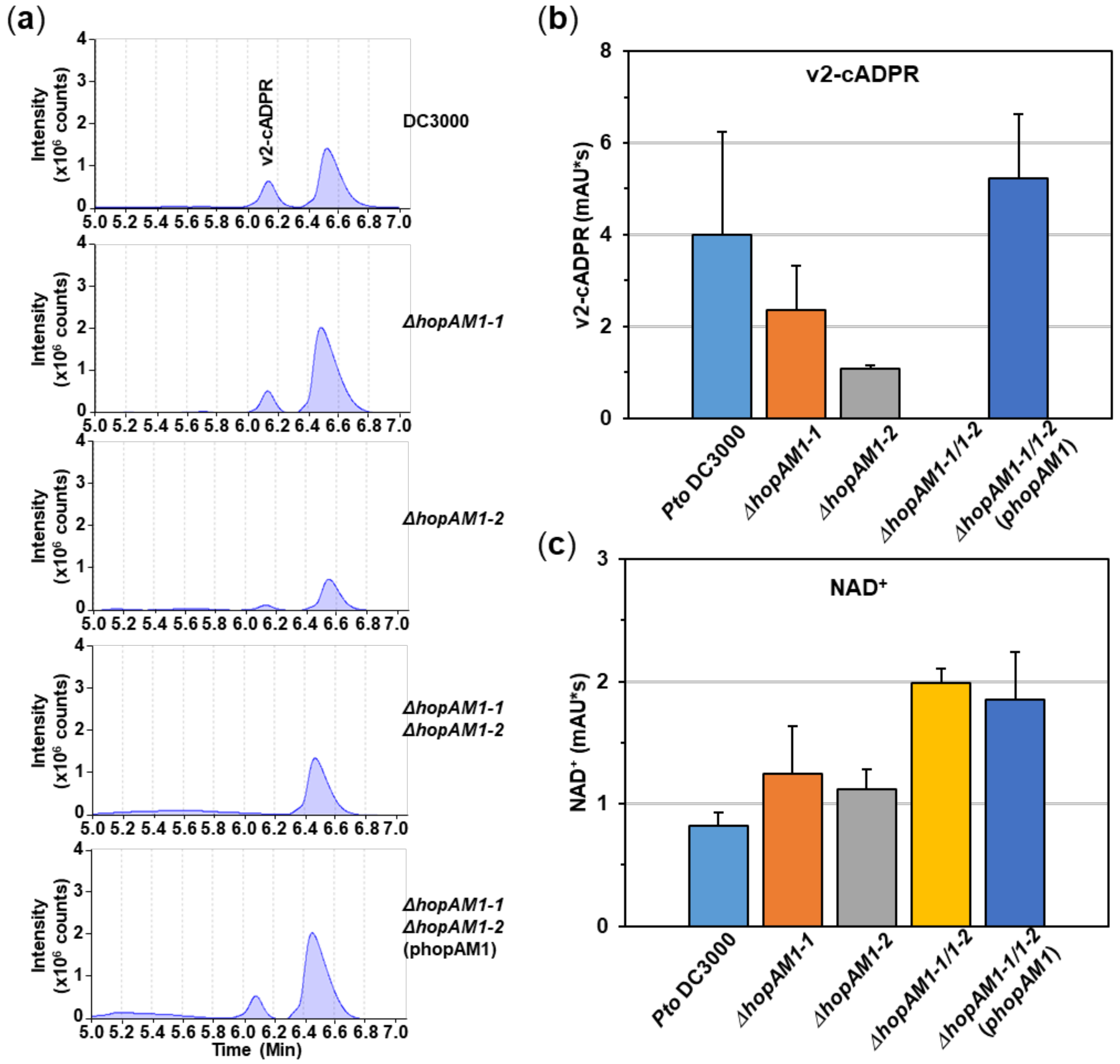

Fig. S5 HopAM1 in *Pto* DC3000 is required to produce v2-cADPR in Arabidopsis. (**a**) HPLC analysis of metabolites in Arabidopsis Col-0 leaves inoculated with *Pto* DC3000, *ΔhopAM1-1*, *ΔhopAM1-2*, *ΔhopAM1-1 ΔhopAM11-2* double mutant, and *ΔhopAM1-1/1-2*(*phopAM1*). (**b**) Quantification of v2-cADPR and (**c**) NAD$^+$ from samples examined in (**a**). Leaves infected with *Pto* DC3000 and complementing strains produced significantly higher amount of v2-cADPR, while v2-cADPR was undetectable in samples infected with *hrcC* and *ΔhopAM1-1/1-2* double mutant. Bars indicate standard error.

Fig. S6 HopAM1's ETI-like response in Arabidopsis Xan-5 is dependent on its conserved residues in TIR domain.

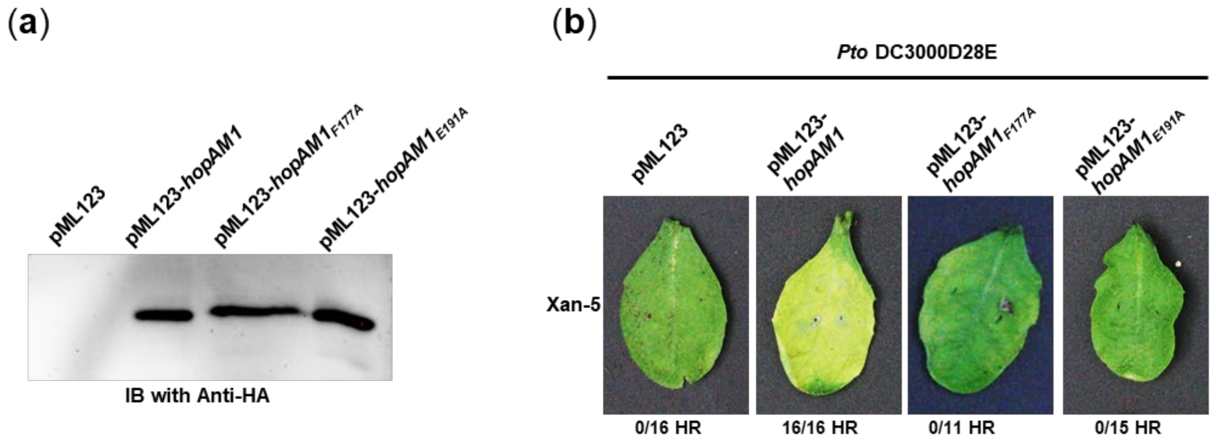

Fig. S6 HopAM1's ETI-like response in Arabidopsis Xan-5 is dependent on its conserved residues in TIR domain. (**a**) Immunoblot detection of expression of HopAM1 and its mutants in the indicated *Pto* DC3000D28E strains. (**b**) ETI-like response of HopAM1 and its mutants in Arabidopsis Xan-5. *Pto* DC3000D28E strains with indicated plasmids carrying respective plasmids were infiltrated into Arabidopsis Xan-5 leaves at cell density of 2×10^8 cells ml^{-1}. Images were taken 3 days post inoculation.

Fig. S7 HopAM1 contributes to *Pto* DC3000 virulence in tomato plants in a manner dependent on its putative catalytic residue.

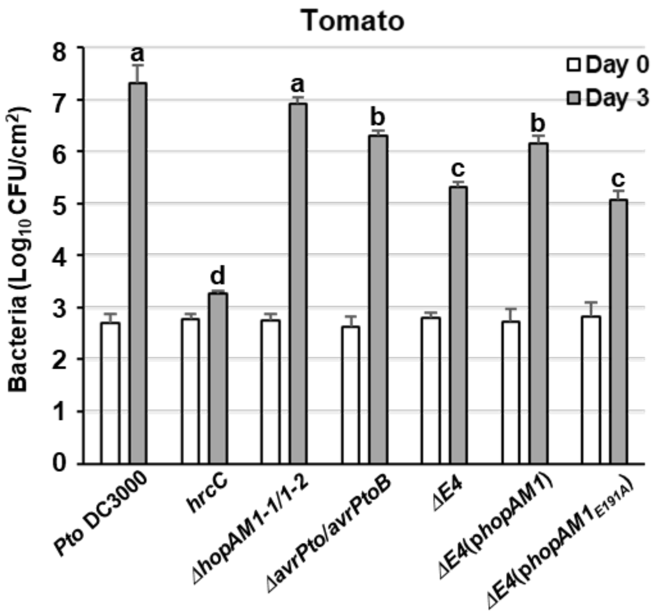

Fig. S7 HopAM1 contributes to *Pto* DC3000 virulence in tomato plants in a manner dependent on its putative catalytic residue. Tomato cv. "Money Maker" plants were spray-inoculated at $2x10^8$ cells ml^{-1} with *Pto* DC3000, *hrcC* type-III defective mutant, double mutant *ΔhopAM1-1 ΔhopAM1-2* (*ΔhopAM1-1/1-2*), double mutant *ΔavrPto ΔavrPtoB*, and a quadruple mutant *ΔavrPto ΔavrPtoB ΔhopAM1-1 ΔhopAM1-2* (*ΔE4*) and the latter complemented with wild type *hopAM1* or TIR-domain mutant derivative *hopAM1*$_{E191A}$. After 0 and 3 days, leaf tissue was assayed for bacterial growth. Bacterial growth assays were repeated 3 times with similar results. Bars denote standard error. Letters denote statistical significance ($p < 0.05$).

Fig. S8 Mutation of conserved residues in HopAM1's TIR domain do not affect its subcellular localization

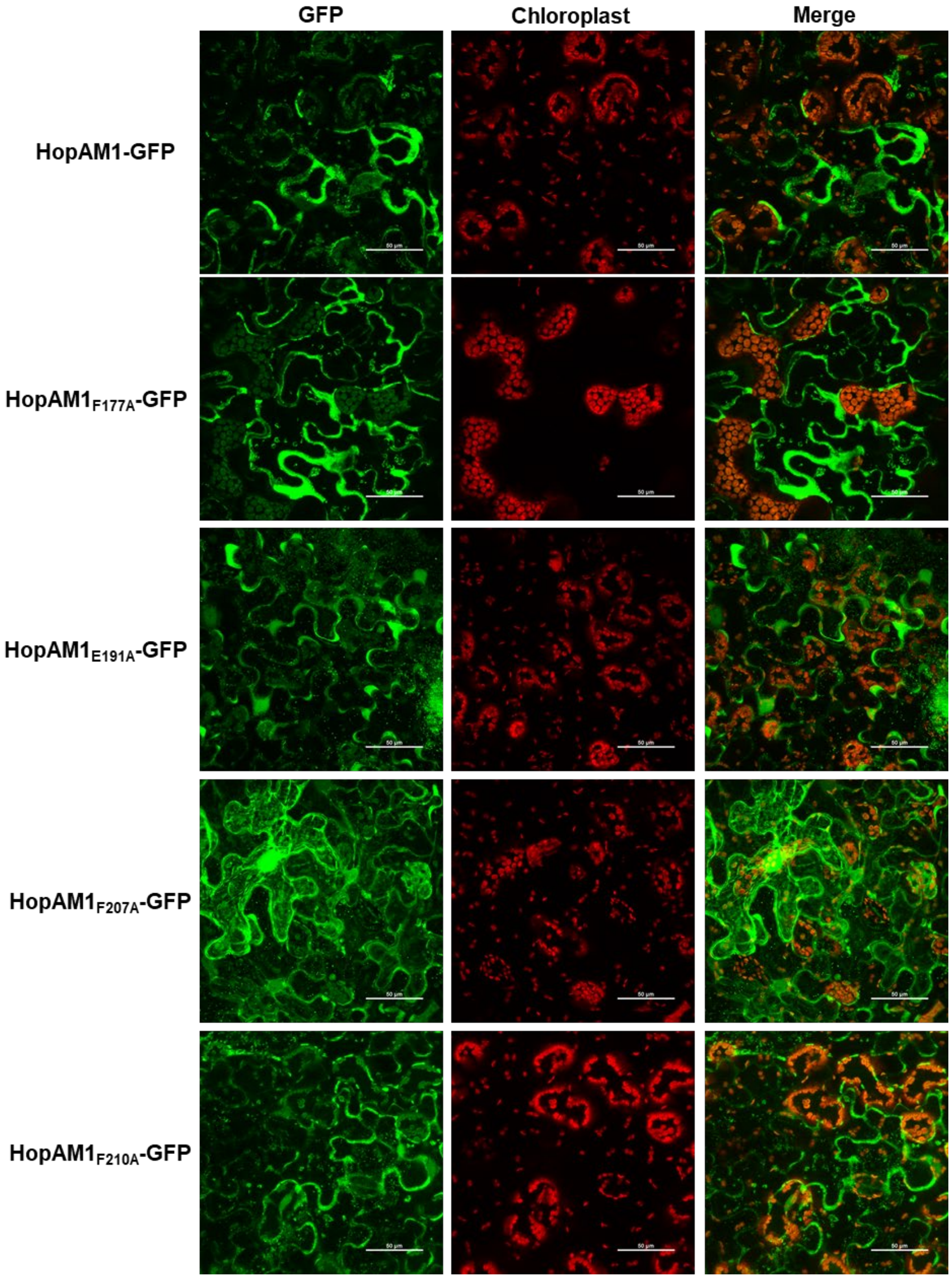

Fig. S8 Mutation of conserved residues in HopAM1's TIR domain do not affect its subcellular localization. GFP fusion proteins of HopAM1 and the respective mutation derivatives were visualized with a confocal microscope. The GFP fusions were transiently expressed via *Agrobacterium*-mediated transformation in *N. benthamiana*. Images were captured at 40x magnification. Scale bars = 50 μm.

Table S1 Strains and plasmids used in this study

Strains or plasmids	Characteristics	References or sources
Pseudomonas syringae pv. *tomato* DC3000	Wild type; spontaneous Rif^R	(Cuppels, 1986)
hrcC	*hrcC* mutant defective in T3SS, Cm^R	(Yuan & He, 1996)
JSU2	*ΔhopAM1-1 ΔhopAM1-2*, *Pto* DC3000 *hopAM1-1 hopAM1-2* double mutant, Rif^R	This study
JSU7	*ΔavrPto ΔavrPtoB*, *Pto* DC3000 *avrPto avrPtoB* double mutant, Rif^R	This study
JSU8	*ΔhopAM1-1 ΔhopAM1-2 ΔavrPto ΔavrPtoB*, *Pto* DC3000 *hopAM1-1 hopAM1-2 avrPto avrPtoB* quadruple mutant (*ΔE4*), Rif^R	This study
Pto DC3000*D28E*	*Pto* DC3000 mutant lacking 28 effector genes, Rif^R, Sp^R, Gm^R	(Cunnac *et al.*, 2011)
Pto DC3000*D28E hrcU*	*Pto* DC3000 mutant lacking *hrcU* and 28 effector genes, Rif^R , Sp^R Gm^R	(Cunnac *et al.*, 2011)
Pseudomonas fluorescens 55	Nx^R	M. Sasser
E. coli		
DH5α	*supE44 ΔlacU169(φ80lacZΔM15) hsdR17 recA1 endA1 gyrA96 thi-1 relA1*, Nal^R	(Hanahan, 1983)<br>Life Technologies
BL21	*fhuA2 [lon] ompT gal [dcm]* Δ*hsdS*	Novagen
DB3.1	*F⁻ gyrA462 endA1* Δ*(sr1-recA) mcrB mrr hsdS20* (*r*B⁻, *m*B⁻) supE44 ara-14 galK2 lacY1 proA2 rpsL20 (Sm^R) *xyl-5* λ⁻ *leu mtl-1*	Invitrogen
Agrobacterium tumefaciens C58C1	Rif^R, Gm^R	
Yeast		
Saccharomyces cerevisiae EGY48	*MATa, his⁻, trp1⁻, ura3⁻, LexAop(x6)-LEU2*	Clonetech
pER8	Binary vector with estradiol-inducible expression, Sp^R	(Zuo *et al.*, 2000)
pET28a(+)	HIS-tag protein expression vector, Km^R	Novagen
pGilda	Yeast expression Lex-A fusion vector, Ap^R His⁻	Clontech
pHIR11	Cosmid carrying a full functional T3SS and effector *hopA1* derived from *P. syringae* pv. *syringae* 61, Tc^R	(Alfano *et al.*, 1997)

pK18mobsacB	*sacB*-based vector for bacterial mutagenesis	(Schäfer *et al.*, 1994)
pK7FWG2	Gateway destination binary vector with GFP fusion at C-terminus, Sp^R Cm^R	(Karimi *et al.*, 2002)
pLN462	pZP212-derived P35S-gateway-HA-T35S destination vector, Sp^R Cm^R	(Jamir *et al.*, 2004)
pLN507	pGilda-hopAM1, pGilda derivative carrying hopAM1, Ap^R	(Jamir *et al.*, 2004)
pLN533	pGilda-hopA1$_{61}$, pGilda derivative carrying hopA1$_{61}$, Ap^R	This study
pLN565	pGilda-Bax, pGilda derivative carrying Bax, Ap^R	(Jamir *et al.*, 2004)
pLN604	pER8-derived Gateway-HA destination vector, Sp^R Cm^R	(Guo *et al.*, 2009)
pLN615	pML123-derived gateway-HA destination vector, Gm^R Cm^R	(Guo *et al.*, 2009)
pLN1965	pHIR11 derivative carrying *hopA1* deletion, Tc^R Sp^R	(Guo *et al.*, 2009)
pLN5841	pK18mobsacB derived Gateway destination vector; Cm^R, Km^R	This study
pLN6374	pML123-hopAM1$_{F210A}$, pLN615 derivative carrying hopAM1$_{F210A}$-HA, Gm^R	This study
pLN6375	pER8-hopAM1, estradiol inducible pLN604 derivative carrying hopAM1-HA, Sp^R	This study
pLN6381	pML123-hopAM1, pLN615 derivative carrying hopAM1-HA, Gm^R	This study
pLN6382	pML123-hopAM1$_{F177A}$, pLN615 derivative carrying hopAM1$_{F177A}$-HA, Gm^R	This study
pLN6383	pML123-hopAM1$_{F207A}$, pLN615 derivative carrying hopAM1$_{F207A}$-HA, Gm^R	This study
pLN6384	p35S-hopAM1, pLN462 derivative carrying hopAM1-HA, Sp^R	This study
pLN6385	p35S-hopAM1$_{F177A}$, pLN462 derivative carrying hopAM1$_{F177A}$-HA, Sp^R	This study
pLN6387	p35S-hopAM1$_{F207A}$, pLN462 derivative carrying hopAM1$_{F207A}$-HA, Sp^R	This study
pLN6388	p35S-hopAM1$_{F210A}$, pLN462 derivative carrying hopAM1$_{F210A}$-HA, Sp^R	This study
pLN6572	p35S-hopAM1-GFP, pK7FWG2-derivative carrying hopAM1-GFP, Sp^R	This study
pLN6573	p35S-hopAM1$_{F177A}$-GFP, pK7FWG2-derivative carrying hopAM1$_{F177A}$, Sp^R	This study
pLN6576	p35S-hopAM1$_{F207A}$-GFP, pK7FWG2-derivative carrying hopAM1$_{F207A}$-GFP, Sp^R	This study
pLN6650	pET28a(+)-*hopAM1$_{E191A}$-His,* pET28a(+) derivative carrying *hopAM1$_{E191A}$*, Km^R	This study
pLN6651	pET28a(+)-*hopAM1-His,* pET28a(+) derivative carrying *hopAM1*, Km^R	This study
pLN6656	pGilda-hopAM1$_{E191A}$, pGilda derivative carrying hopAM1$_{E191A}$, Ap^R	This study

pLN6663	pER8-hopAM1$_{E191A}$, estradiol inducible pLN604 derivative carrying hopAM1$_{E191A}$-HA, Sp^R	This study
pLN6664	pML123-hopAM1$_{E191A}$, pLN615 derivative carrying hopAM1$_{E191A}$-HA, Gm^R	This study
pML123	Broad-host-range vector, Gm^R Km^R	(Labes *et al.*, 1990)
pMQ1	pLN5841 derivative carrying the upstream/downstream of *avrPto*	This study
pMQ2	pLN5841 derivative carrying the upstream/downstream of *hopAM1-1*	This study
pMQ3	pLN5841 derivative carrying the upstream/downstream of *hopAM1-2*	This study
pMQ4	pLN5841 derivative carrying the upstream/downstream of *avrPtoB*	This study
pMQ11	pML123-hopAM1$_{I168A}$, pLN615 derivative carrying hopAM1$_{I168A}$-HA, Gm^R	This study
pMQ12	p35S-hopAM1$_{E191A}$, pLN462 derivative carrying hopAM1$_{E191A}$-HA, Sp^R	This study
pMQ13	p35S-hopAM1$_{F210A}$-GFP, pK7FWG2-derivative carrying hopAM1$_{F210A}$-GFP, Sp^R	This study
pMQ14	p35S-hopAM1$_{E191A}$-GFP, pK7FWG2-derivative carrying hopAM1$_{E191A}$-GFP, Sp^R	This study
pZG-19	pGilda-hopAM1$_{F207A}$, pGilda derivative carrying hopAM1$_{F207A}$, Ap^R	This study
pZG-20	pGilda-hopAM1$_{F177A}$, pGilda derivative carrying hopAM1$_{F177A}$, Ap^R	This study
pZG-21	pGilda-hopAM1$_{E197A}$, pGilda derivative carrying hopAM1$_{E197A}$, Ap^R	This study
pZG-22	pGilda-hopAM1$_{F210A}$, pGilda derivative carrying *hopAM1*$_{F210A}$, Ap^R	This study
pAbTIR	pET30 derivative carrying AbTIR, Km^R	This study

Methods S1 Supporting information for Materials and Methods

Bacterial cultures and growing conditions

E. coli strains DH5α, DB3.1, and BL21 were grown on LM medium amended with selective antibiotics at 37°C. *A. tumifaciens* C58C1 strains were maintained on LM medium with antibiotics at 30°C. *P. syringae* and *P. fluorescens* strains were maintained on King's B (KB) (King *et al.*, 1954) medium with antibiotics at 30°C. All strains used in this study are listed in supplementary Table S1. Antibiotics were used at the following concentrations (mg ml^{-1}): ampicillin, 100; Chloramphenicol, 20; gentamycin, 10; kanamycin, 50; nalidixic acid, 20; rifampicin, 100; spectinomycin 50; and tetracycline, 20.

DNA manipulation

Desired DNA regions were amplified from *Pto* DC3000 genomic DNA using DreamTaq polymerase (Thermo Fisher Scientific). Standard protocols were followed for conventional ligation or gateway cloning. For conventional cloning, PCR products were digested with restriction enzymes and ligated to vectors with T4 DNA Ligase (New England Biolabs). For gateway cloning, amplicons were cloned into a pENTR vector (Invitrogen) and the resulting pENTR plasmids with insertion were recombined into gateway destination vectors with LR clonase to create recombinant constructs. Site-directed mutagenesis of the *hopAM1* gene was achieved by amplifying two overlapping fragments of *hopAM1* by PCR using primers introduced with specific mutation, then fusing the fragments in a second round of PCR with *hopAM1* specific forward and reverse primers. The resulting PCR products were cloned into pENTR and the constructs containing desired mutation were confirmed by sequencing. All constructs generated for this study are listed in supplementary Table S1.

Purification of recombinant protein

Overnight *E. coli* BL21 cultures carrying pET28a(+) plasmids that express *hopAM1*, $hopAM1_{E191A}$, as well as empty vector (mock) were diluted at a ratio of 1:50 into 100 ml LM and grown with shaking at 37°C till an optical density (OD_{600nm}) of 0.6 (~4 hours). Cultures were induced with 1 mM IPTG (Invitrogen) for 2.5 hours at 37°C. The induced cultures were pelleted by centrifugation at 16,000 g and resuspended with 10 ml CelLytic B (Sigma-Aldrich) buffer amended with Benzonase (50 U ml^{-1}) (Sigma-Aldrich), Lysozyme (200 µg ml^{-1}) (Sigma-Aldrich), and Protease Inhibitor Cocktail for His-tagged protein purification (10 µl ml^{-1}) (Sigma-Aldrich). Cells were agitated at room temperature (25°C) for 15 minutes, then centrifuged at 16,000 g for 10 minutes. The supernatant cell lysates were mixed with 200 µl HIS-select Nickle Affinity resin (Sigma-Aldrich) for 15 minutes at room temperature (25°C) with agitation. The resin was washed three times with 2 ml 50 mM sodium phosphate, pH 8.0, 0.3 M sodium chloride and then eluted in 4 ml 50 mM sodium phosphate, pH 8.0, 0.3 M sodium chloride, and 250 mM imidazole. Eluate was concentrated for 45 minutes at 4,000 g using an Amicon Ultracel 3K Centrifugal Filter (Millipore) and then mixed with 2 ml of 0.64x PBS and re-concentrated two more times to a final volume of 100 µL.

Proteins and immunoblot analysis

For plant protein extraction, 48 hours after infiltration leaves were sampled with a 16 mm diameter cork borer. Samples were frozen in liquid nitrogen and ground with a plastic pestle, then resuspended in 200 µL x1.5 sample buffer, vortexed and boiled for 10 minutes before storage at -20°C until SDS-PAGE.

For yeast protein extraction, 1 ml of culture was spun down and resuspended in 0.1 M NaOH for five minutes as described by (Kushnirov, 2000), then spun down for 30 seconds at

15,000 g and resuspended in 100 µL x1.5 sample buffer, vortexed and boiled for 10 minutes before storage at -20°C until SDS-PAGE.

Extracted proteins were separated by SDS-PAGE and transferred to PVDF membrane using a TransBlot Turbo transfer system (Bio-RAD) with the standard setting. Immunoblots were performed with appropriate antibodies following standard protocols. The following primary antibodies were used: Rabbit anti-Lex (Millipore), Rat anti-HA (Roche), and Mouse anti-HIS (Sigma-Aldrich). All secondary antibodies were conjugated with alkaline phosphatase (Sigma-Aldrich). Immunoblots were visualized using CDP-Star (Roche).

Unmarked mutagenesis of *P. syringae* DC3000

Upstream and downstream DNA regions of *avrPto*, *avrPtoB*, *hopAM1-1*, or *hopAM1-2* were PCR-amplified using DreamTaq or Phire II DNA polymerase (Thermo Fisher Scientific) and cloned into a gateway pENTR vector (Invitrogen). The resulting pENTR constructs were recombined with pLN5841 by LR clonase creating sacB-based constructs for unmarked mutagenesis. The resulting mutagenesis constructs were conjugated using tri-parental or bi-parental mating and integrated into the chromosome of *Pto* DC3000 by homologous recombination. Desired deletions were screened on KB medium containing sucrose (5%) to counter-select for the survived colonies that lack of antibiotic resistance. The final mutants were further verified by PCR.

Bioinformatics tools

BLAST at NCBI was routinely used for searching homologs of HopAM1. Structural similarity of HopAM1 was predicted using 3-D the threading online tool PHYRE2 (http://www.sbg.bio.ic.ac.uk/~phyre2) (Kelley *et al.*, 2015). Alignments of multiple proteins were made with Clustal Omega with manual correction (https://www.ebi.ac.uk/Tools/msa/clustalo/) (Madeira *et al.*, 2019). MEGA-X was used to build phylogenic trees (Kumar *et al.*, 2018).

Yeast cell death assay

Yeast strain EGY48 carrying pGilda derivatives were grown overnight at 30 °C in synthetic dropout glucose media lacking histidine (glucose-His). The cells were washed and resuspended in ddH_2O to an OD_{600nm} of 0.1. A 10-fold dilution series was plated on glucose-His agar or galactose-His and grown at 30 °C. Colonies were assessed for survival after three days.

Plant materials

All Arabidopsis plants were grown at 24 °C with a 10 hour light/14 hour dark cycle in micro-climate controlled growth chambers. *N. benthamiana* and *N. tabacum* cv. Xanthi plants were grown in standard greenhouses.

***Agrobacterium*-mediated transient assays**

For assays with tobacco, *Agrobacterium tumefaciens* C58C1 strains carrying binary vectors were grown overnight, centrifuged at 4,696 g for 10 min and resuspended in induction medium (0.5 g L^{-1} sodium citrate, 10 mM MES, 1 g L^{-1} ammonium sulfate, 250 µM magnesium sulfate, 0.1% glycerol, 0.2% glucose, 10.5 g L^{-1} potassium phosphate dibasic, 4.5 g L^{-1} potassium phosphate monobasic) containing 150 µM acetosyringone (Fisher Scientific) and incubated with shaking at 30 °C for 6 hours. The induced *Agrobacterium* cultures were pelleted by centrifugation at 4,696 g for 10 min and resuspended in infiltration medium (2.15 g L^{-1} MS basal salts, 10 mM MES, pH5.6) containing 300 µM acetosyringone adjusted to an OD_{600nm} of 0.8. Leaves of *N. tabacum* cv. Xanthi or *N. benthamiana* were infiltrated with needless syringe and plants were kept at room temperature. For inducible-expression constructs, twenty-four hours after infiltration infiltrated leaves were sprayed with 20 µM estradiol (Sigma-Aldrich)

containing 0.02% Silwet-L77 (Lehle Seeds) using a spray bottle. Samples were harvested at indicated time points for metabolite analysis and immunoblot analyses.

Confocal microscopy

GFP fusion proteins were transiently expressed in *N. benthamiana* via *Agrobacterium tumefaciens* infiltration. After 48 hours infiltrated leave discs were detached to monitor green fluorescence and imaged at 40x magnification with a Nikon A1-NiE confocal microscope. Images were visualized using NIS-Elements software with internal ruler.

Plant hypersensitivity response assays

For assays in tobacco, the development of a hypersensitive response was assessed and photographed 72 hours after infiltration with *Agrobacterium* cultures. For assays with Arabidopsis, overnight cultures of *Pto* DC3000 D28E strains were resuspended in 10 mM $MgCl_2$ and adjusted to an OD_{600nm} of 0.1 (equivalent to a cell density of $1x10^8$ CFU ml^{-1}). Leaves of four-week-old *Arabidopsis* accessions Xan-2, Xan-5, or Col-0 plants were infiltrated with a needleless syringe. Plants were covered with a plastic lid and maintained for moisture at room temperature (25°C). The development of a hypersensitive response or symptoms was assessed and photographed after 2-4 days.

***In vitro* NAD hydrolase and enzyme kinetics assays**

Ten microliters of concentrated protein extract were mixed with 3 µL of 250 µM NAD^+ (Selleck Chemicals) in 0.64x PBS and incubated at room temperature (25°C). Samples at 0 and 60 minutes were mixed with 250 µL 50% methanol at -40°C and vortexed, then mixed with 250 µl chloroform at -40°C. Samples were then centrifuged at 15,000 g for 5 minutes at -10°C. The aqueous/methanol layer was removed, lyophilized, and stored at -80°C for HPLC analysis.

To calculate HopAM1 enzyme kinetics, *in vitro* reactions consisting of 10 microliters of protein laden bead suspension and 40 µl of 1, 10, 50, 100, 400, or 600 µM NAD^+ in 25 mM HEPES buffer pH=7.5 at room temperature with constant agitation. At 1 hour, reactions were quenched by pulling the beads to the side and transfer 40 microliters of the reaction mixture to a new tube containing 160 microliters of ice cold 0.5 M HClO4. Ten microliters of 2x Laemmli buffer (BioRad 1610737) supplemented with β-mercaptoethanol were added to the beads and boiled for ten minutes and subjected to gel electrophoresis on a 4-12% gradient Bis-TRIS polyacrylamide gel (Invitrogen NW04125). Protein concentration was approximated by staining the SDS-page gel with Coomassie blue stain (instant blue, AbCam AB119211) and application of densitometry using ImageJ with comparison to a set of BSA standards. NAD^+ consumption rates for HopAM1 were calculated by finding NAD^+ peak area reduction compared to empty vector control and using NAD^+ calibration curve to convert peak area reduction to change in number of moles of NAD^+. Average change in NAD moles per minute of reaction was then calculated. The Curve Fitting Tool on MATLAB (Version R2020a) was used to fit NAD^+ consumption kinetics data to the Michaelis-Menten equation, V = Vmax * (NAD^+ Concentration) / (Km + NAD^+ Concentration) where parameters Vmax and Km are constants. Optimal values were output for the parameters Vmax and Km as well as an R^2 value for the data's fit to the model.

ROS assay

ROS production was determined following a previously described protocol (Asai *et al.*, 2008). Briefly, *Pseudomonas fluorescens* 55 (*Pf*) (pLN1965) strains were grown overnight, suspended in 10 mM $MgCl_2$ and infiltrated with a needleless syringe into leaves of five-week-old *Arabidopsis* plants at a density of $1x10^7$ CFU ml^{-1}. After 24 hours leaf discs were excised using a 4 mm diameter cork borer and incubated in H_2O in white 96 well plate overnight. H_2O is then replaced with 0.5 mM L-012 and 1 µM flg22 in 10 mM MOPS-KOH at pH 7.4. The production of

ROS was determined by counting photons using with a Synergy 5 luminometer (BioTek, Winooski, VT, USA).

Callose deposition assay

Pf(pLN1965) strains were grown overnight, suspended in 10 mM $MgCl_2$ and infiltrated with a needleless syringe into leaves of five-week-old *Arabidopsis* Col-0 plants at a density of $1x10^7$ CFU ml^{-1}. After 48 hours, callose deposits in infiltrated leaves were stained with aniline blue and visualized with a Zeiss Axioplan 2 microscope. The numbers of callose deposits were quantified using ImageJ (http://imagej.nih.gov/ij/) following a procedure previously reported (Guo *et al.*, 2016).

***In planta* bacterial growth assay**

P. syringae strains were grown overnight, resuspended in 10 mM $MgCl_2$ and infiltrated with a needleless syringe into leaves of five-week-old *Arabidopsis* at $1x10^5$ cells ml^{-1} or spray-inoculated onto tomato cultivar 'Moneymaker' plants with 0.02% Silwet-L77 at a density of $1x10^8$ cells ml^{-1}. Plants were maintained with moisture in covered trays at room temperature. A 6 mm diameter cork borer was used to sample inoculated leaves at the indicated time points. The samples were ground in 250 µl sterile ddH_2O using a plastic pestle and 20 µl each of a 10-fold serial dilution series was plated on KB agar and incubated at 30°C. Bacterial colonies were numerated after 2-3 days.

References

Alfano JR, Kim H-S, Delaney TP, Collmer A. 1997. Evidence that the *Pseudomonas syringae* pv. syringae hrp-linked hrmA gene encodes an Avr-like protein that acts in an hrp-dependent manner within tobacco cells. *Molecular Plant-Microbe Interactions* **10**(5): 580-588.

Asai S, Ohta K, Yoshioka H. 2008. MAPK signaling regulates nitric oxide and NADPH oxidase-dependent oxidative bursts in *Nicotiana benthamiana*. *The Plant Cell* **20**(5): 1390-1406.

Cunnac S, Chakravarthy S, Kvitko BH, Russell AB, Martin GB, Collmer A. 2011. Genetic disassembly and combinatorial reassembly identify a minimal functional repertoire of type III effectors in *Pseudomonas syringae*. *Proceedings of the National Academy of Sciences* **108**(7): 2975-2980.

Cuppels DA. 1986. Generation and characterization of Tn5 insertion mutations in Pseudomonas syringae pv. tomato. *Applied and Environmental Microbiology* **51**(2): 323-327.

Guo M, Kim P, Li G, Elowsky CG, Alfano JR. 2016. A Bacterial Effector Co-opts Calmodulin to Target the Plant Microtubule Network. *Cell Host & Microbe* **19**(1): 67-78.

Guo M, Tian F, Wamboldt Y, Alfano JR. 2009. The majority of the type III effector inventory of *Pseudomonas syringae* pv. tomato DC3000 can suppress plant immunity. *Molecular Plant-Microbe Interactions* **22**(9): 1069-1080.

Hanahan D. 1983. Studies on transformation of *Escherichia coli* with plasmids. *J Mol Biol* **166**(4): 557-580.

Jamir Y, Guo M, Oh H-S, Petnicki-Ocwieja T, Chen S, Tang X, Dickman MB, Collmer A, R. Alfano J. 2004. Identification of *Pseudomonas syringae* type III effectors that can suppress programmed cell death in plants and yeast. *The Plant Journal* **37**(4): 554-565.

Karimi M, Inzé D, Depicker A. 2002. GATEWAY™ vectors for Agrobacterium-mediated plant transformation. *Trends in Plant Science* **7**(5): 193-195.

Kelley LA, Mezulis S, Yates CM, Wass MN, Sternberg MJE. 2015. The Phyre2 web portal for protein modeling, prediction and analysis. *Nature Protocols* **10**(6): 845-858.

King EO, Ward MK, Raney DE. 1954. Two simple media for the demonstration of pyocyanin and fluorescein. *Journal of Laboratory Medicine* **22**: 301-307.

Kumar S, Stecher G, Li M, Knyaz C, Tamura K. 2018. MEGA X: molecular evolutionary genetics analysis across computing platforms. *Molecular Biology and Evolution* **35**(6): 1547-1549.

Kushnirov VV. 2000. Rapid and reliable protein extraction from yeast. *Yeast* **16**(9): 857-860.

Labes M, Pühler A, Simon R. 1990. A new family of RSF1010-derived expression and lac-fusion broad-host-range vectors for gram-negative bacteria. *Gene* **89**(1): 37-46.

Madeira F, Park YM, Lee J, Buso N, Gur T, Madhusoodanan N, Basutkar P, Tivey ARN, Potter SC, Finn RD, et al. 2019. The EMBL-EBI search and sequence analysis tools APIs in 2019. *Nucleic Acids Research* **47**(W1): W636-W641.

Schäfer A, Tauch A, Jäger W, Kalinowski J, Thierbach G, Pühler A. 1994. Small mobilizable multi-purpose cloning vectors derived from the *Escherichia coli* plasmids pK18 and pK19: selection of defined deletions in the chromosome of Corynebacterium glutamicum. *Gene* **145**(1).

Yuan J, He SY. 1996. *The Pseudomonas syringae* Hrp regulation and secretion system controls the production and secretion of multiple extracellular proteins. *J Bacteriol* **178**(21): 6399-6402.

Zuo J, Niu QW, Chua NH. 2000. An estrogen receptor‐based transactivator XVE mediates highly inducible gene expression in transgenic plants. *The Plant Journal* **24**(2): 265-273.
